# Hydrogel Electrolytes-Based Rechargeable Zinc-Ion Batteries under Harsh Conditions

**DOI:** 10.1007/s40820-025-01727-y

**Published:** 2025-04-22

**Authors:** Zhaoxi Shen, Zicheng Zhai, Yu Liu, Xuewei Bao, Yuechong Zhu, Tong Zhang, Linsen Li, Guo Hong, Ning Zhang

**Affiliations:** 1 College of Chemistry and Materials Science, Key Laboratory of Analytical Science and Technology of Hebei Province, Institute of Life Science and Green Development, Hebei University, Baoding, 071002 People’s Republic of China; 2 Department of Materials Science Engineering & Centre of Super-Diamond and Advanced Films (COSDAF), City University of Hong Kong, Kowloon, Hong Kong, 999077 People’s Republic of China

**Keywords:** Hydrogel electrolytes, Rechargeable zinc-ion batteries, Harsh conditions, Design strategies, Energy storage

## Abstract

Rechargeable zinc (Zn)-ion batteries (RZIBs) with hydrogel electrolytes (HEs) have gained significant attention in the last decade owing to their high safety, low cost, sufficient material abundance, and superb environmental friendliness, which is extremely important for wearable energy storage applications. Given that HEs play a critical role in building flexible RZIBs, it is urgent to summarize the recent advances in this field and elucidate the design principles of HEs for practical applications. This review systematically presents the development history, recent advances in the material fundamentals, functional designs, challenges, and prospects of the HEs-based RZIBs. Firstly, the fundamentals, species, and flexible mechanisms of HEs are discussed, along with their compatibility with Zn anodes and various cathodes. Then, the functional designs of hydrogel electrolytes in harsh conditions are comprehensively discussed, including high/low/wide-temperature windows, mechanical deformations (e.g., bending, twisting, and straining), and damages (e.g., cutting, burning, and soaking). Finally, the remaining challenges and future perspectives for advancing HEs-based RZIBs are outlined.

## Highlights

- The developing history and recent advances of hydrogel electrolytes for rechargeable zinc-ion batteries under harsh conditions are summarized.
- The fundamentals, species, and mechanisms of the hydrogel electrolytes are discussed.
- The functional design strategies for advanced hydrogel electrolytes under harsh conditions are discussed.
- The remaining challenges and future perspectives for the practical application of hydrogel electrolyte-based rechargeable zinc-ion batteries are discussed.

## Graphical abstract

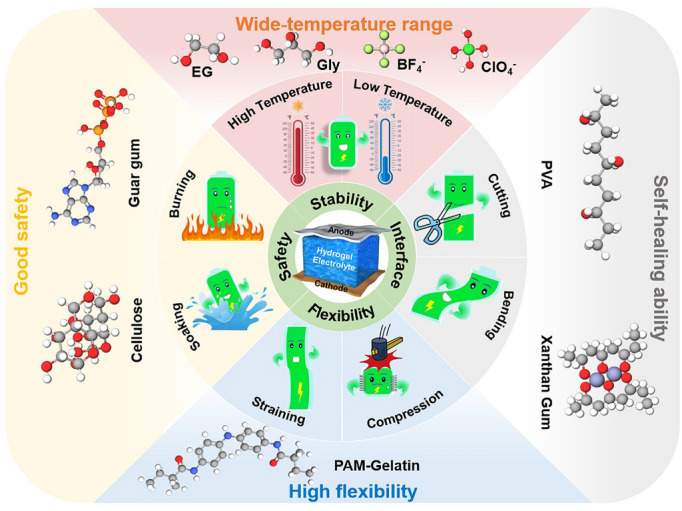

## Introduction

The flourishing development of flexible and wearable electronics has spawned a huge demand for efficient energy storage devices [1, 2]. Commercial lithium-ion batteries (LIBs) are suffering from flammable organic electrolytes despite their sophisticated technologies and dominant market in power resources [3]. Recently, rechargeable zinc-ion batteries (RZIBs) have attracted fast-growing interest in various energy storage applications, benefiting from the high safety, low cost, and eco-friendliness of water systems [3–5]. Moreover, the Zn metal anode possesses natural abundance, facile manufacturing, nonflammability in water, and a desirable theoretical volume capacity (820 mAh g^−1^ or 5855 mAh cm^−3^) [6]. Nevertheless, the Zn anode suffers from poor reversibility because of the undesired Zn dendrite growth and water-induced side reactions (e.g., H_2_ evolution and Zn corrosion) [7]. Moreover, the traditional oxide-based cathode materials (e.g., V_2_O_5_ and MnO_2_) are easily dissolved in aqueous electrolytes, leading to inferior electrochemical performance of batteries [8–10]. Besides, the aqueous electrolytes endure a narrow voltage window along with hydrogen/oxygen evolution reaction (HER and OER), resulting in continuous consumption of electrolytes and battery failure [11–13].

Hydrogel electrolytes (HEs) are pivotal in developing high-performance RZIBs, as they effectively address the limitations of aqueous electrolytes [14–18]. Firstly, HEs inhibit Zn dendrite formation by modifying the anode surface [4]. Secondly, their quasi-solid nature reduces fluidity, thereby mitigating cathode material dissolution. Furthermore, water-saturated HEs reduce the reactivity of free water, expanding the voltage window and minimizing water-induced side reactions [19, 20]. Notably, hydrogels can serve multiple roles in RZIBs, acting as electrolytes, separators, protective layers, active materials, and binders, thanks to their mechanical strength, self-healing ability, hydrophilicity, and wide-temperature adaptability [21].

To date, massive efforts have been made to develop HEs for RZIBs to adapt to different scenarios [22–31]. For instance, cellulose, polyvinyl alcohol (PVA), and polyhydric additives have been employed to improve the electrochemical performance of HEs-based RZIBs in a wide temperature range of −70 to 100 °C [22–24]; polyacrylamide (PAM), cooperating with gelatin, cellulose, and chitosan, has served as HEs with good mechanical strength to resist diverse deformations and damages [25–27]; the ultra-hyperelastic and highly entangled hydrogel species have been designed for relieving swelling effect [28, 29]; and the additives of flame retardants have been introduced into HEs to prevent combustion of batteries [30, 31]. However, the harsh conditions of extreme temperatures, mechanical deformations, and damages will bring out the negative effects on the electrode–electrolyte interface stability, ionic conductivity, and HEs integrity, seriously influencing the electrochemical performance and lifespan of hydrogel-based RZIBs. To the best of our knowledge, the recent advances involving functional categories and design strategies for HEs under harsh conditions have rarely been summarized systematically.

Hence, we comprehensively review the hydrogel selection and construction to achieve high-performance RZIBs under harsh conditions. In the first part of this review, the species and characters, developing history, current progress, and electrolyte–electrode compatibility of HEs are discussed in detail: The hydrogel polymers are mainly categorized into natural species (e.g., gelatin, cellulose) and synthetic species (e.g., PAM, PVA); the development history focuses on the reports of HEs-based RZIBs in recent one decade, from the emerging to blooming; and the hydrogel compatibility with electrodes is highlighted with key interface issues, as well as the solutions for overcoming Zn dendrites, hydrogen evolution reaction (HER), cathodes dissolution. In the second part, this review emphasizes the functional design strategies toward HEs-based RZIBs under low/high temperatures, mechanical deformations (i.e., straining, compression, bending, and twisting), and severe damages (i.e., cutting, burning, and soaking). Meanwhile, this section reviews the physical, chemical, and electrochemical performances of RZIBs under such harsh conditions. The final part outlines the potential practical applications of HEs in flexible and wearable electronic devices and future perspectives to build better HEs-based RZIBs.

## Material Fundamentals

Hydrogels are cross-linked polymeric materials with 3D network structures comprised of polymer chains and inner enclosed water [22]. Based on many studies, it is believed that two key roles determine the properties of hydrogel: monomer and cross-linking method. Monomer controls the types and intrinsic characters of hydrogel, providing various chemical bonds and hydrophilic functional groups (e.g., hydroxyl, amino, and carboxyl groups) for the cross-linking of hydrogels [18]. Different cross-linking method regulates the cross-linking degrees of hydrogel network and covalent chemical/physical bonding, which is also crucial in affecting the hydrogel properties. Typically, high degree cross-linking will enhance the mechanical strength of hydrogels.

**Fig. 1:**
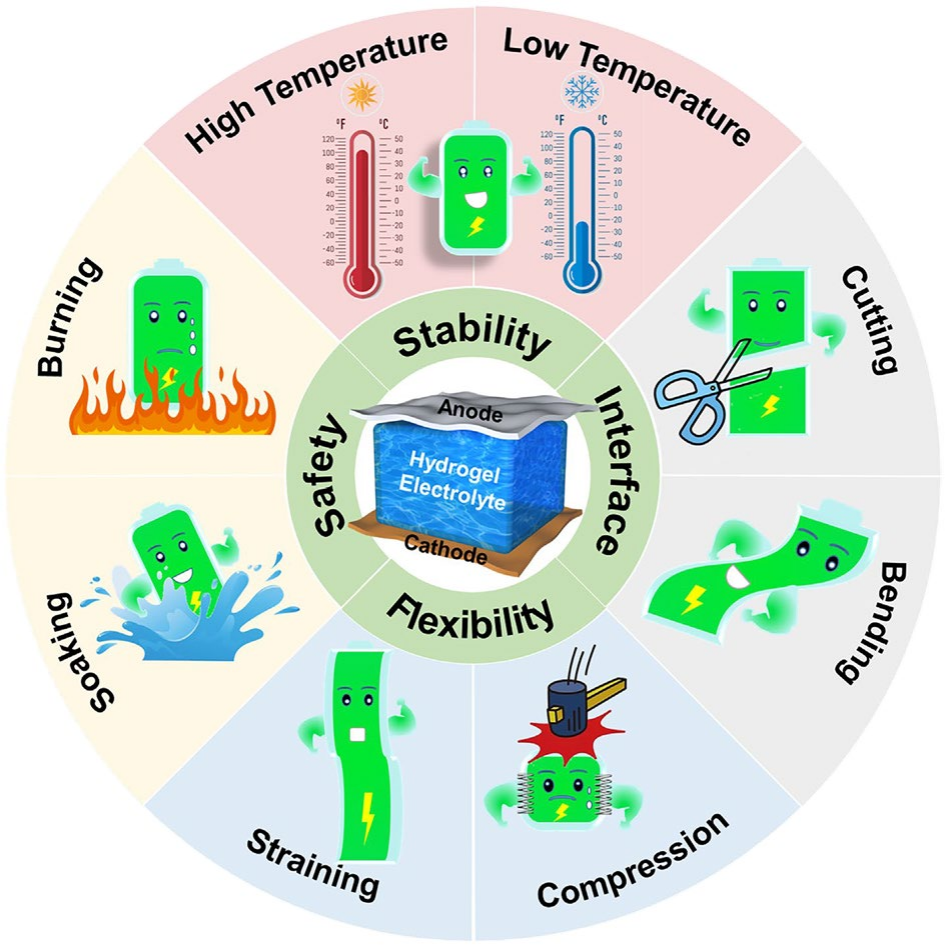
Schematic diagram and advantages of the RZIBs based on HEs (central part) under various harsh conditions (outer ring)

Generally, the hydrogels perform good safety, flexibility, hydrophilicity, ionic conductivity, mechanical strength, thermal and chemical stabilities, and excellent interface compatibility with electrodes, which are the prerequisites as electrolytes. Figure 1 illustrates the schematic diagram of RZIBs based on hydrogel electrolytes, highlighting their advantages under various harsh conditions. Most HEs-based works have studied the performances of RZIBs under extreme temperatures, mechanical deformations, and damages. Referring to the properties and specific criteria of hydrogel polymers in these works, suitable hydrogel species can be selected as the quasi-solid-state electrolytes to improve the performance of rechargeable RZIBs in poor environments. Hence, this section first categorizes the diverse hydrogel species with different characters, then introduces the research history of HEs-based RZIBs, and finally discusses the interface compatibility between HEs and anode/cathode.

### Species of Hydrogel Electrolytes

#### Mono-Component Species

**Fig. 2:**
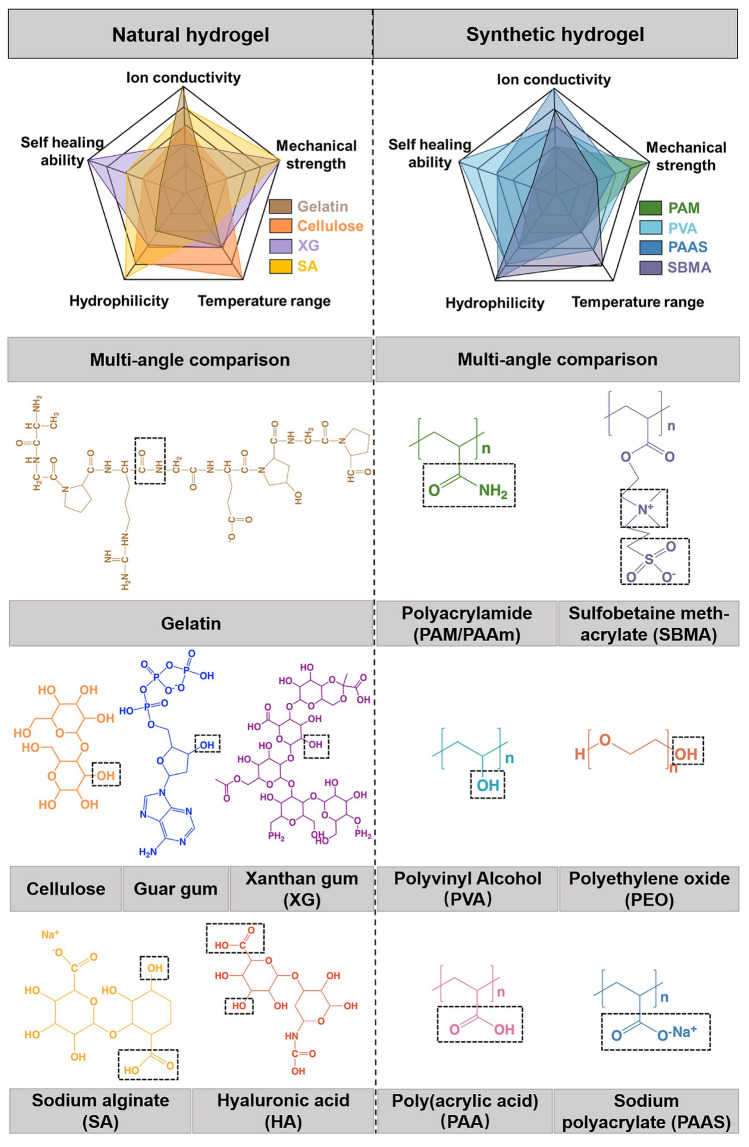
Natural hydrogel species with their multi-angle comparison: gelatin, guar gum, xanthan gum, cellulose, sodium alginate, and hyaluronic acid; and synthetic hydrogel species with their multi-angle comparison: polyacrylamide, polyacrylic acid, polyvinyl alcohol, polyethylene oxide, sodium polyacrylate, and sulfobetaine methacrylate

Mono-component hydrogel species are mainly classified into two categories (Fig. 2): (1) natural hydrogels, e.g., gelatin, guar gum, xanthan gum, cellulose, etc.; (2) synthetic hydrogel cross-linked by physical/chemical composite approaches, e.g., PAM, PVA, PAA, etc. The five-angle comparison graphs are provided to clarify the merits and defects of each natural or synthetic hydrogel. The natural species generally have advantages in higher hydrophilicity, wider temperature windows, and better biocompatibility, while the synthetic species have advantages in stronger mechanical strength, better self-healing ability, and higher ion conductivity. For example, gelatin-based HEs possess good biocompatibility and temperature adaptability due to the abundant –CONH_2_ groups, but their mechanical strength is generally low; PAM-based HEs feature excellent flexibility because the regular network with H-bond interaction can enhance the mechanical strength, but they generally suffer from poor biocompatibility and interfacial compatibility.

*Gelatin* Gelatin is one kind of macromolecular and hydrophilic helical hydrogel composed of three types of chains in a converged network, which contains plenty of hydrogens, amidogens, carbonyls, and other hydrophilic groups. These groups in gelatin are beneficial to the fluidity of ions, further enhancing the ion conductivity in HEs. Han et al. fabricated the self-standing gelatin-based HEs and applied them into the quasi-solid-state Zn batteries, together with the Zn anode and the LiMn_2_O_4_ cathode. It was found that this kind of HEs presented a high ion conductivity of 6.15 × 10^−3^ S cm^−1^ and intimate contact of interface with both cathode and anode [32]. Benefitting from excellent compatibility of interface, the hydrogel of gelatin exhibits high specific capacity, excellent Coulombic efficiency (CE), and stable charge–discharge cycles. Zhu et al. modified the interface between gelatin and electrodes to increase the device energy density of the Zn–MnO_*x*_ microbatteries to 21 mWh cm^−3^, maintain their CE of 100%, and prevent performance failure from cutting, bending, twisting, crimping, and submerging in water [33]. Besides, the gelatin can widen the working temperature ranges when serving in flexible, electrochromic, and rechargeable Zn//polypyrrole (PPy) batteries as electrolytes [28]. Such batteries can deliver a high capacity and perform a stable operation under 25 ~ 80 °C.

*Guar gum* Guar gum is a non-toxic, renewable, cost-effective, hydrophilic, and macromolecular hydrogel polymer species that can be extensively applied in flexible batteries or supercapacitor devices [34, 35]. As shown in Fig. 2, guar gum presents an ordered structure of *α*, *β*-1, 4-mannose chain interposed with *α*-1, 6-galactose substituents in every two units. Benefiting from the uniform poly-anion and hydroxyl groups (–OH), guar gum was employed as HEs in numerous works about RZIBs: By combustion testing, Huang et al. demonstrated guar gum with remarkable fire-resistant properties, which was attributed to the nonflammable –OH groups in molecules [36]; According to the results of electrochemical characterization, Xu et al. showed guar gum with a high ion conductivity and specific capacity since the abundant –OH and poly-anion groups enhanced the ions fluidity [34]; based on the measurement of temperature adaptability, Wang et al. reflected the water retention capacity in high temperature and anti-freezing properties in low temperature owing to the effects of a large amount of H-bonds and –OH groups.

*Xanthan Gum* Xanthan gum is another kind of natural polymer structure with a large and complex exopolysaccharide, consisting of *α*, *β*-1, 4-linked glucan backbone, and trisaccharide side chains [37]. The alternating d-glucosyl groups on the side chains contribute to high salt tolerance; the rich –OH groups enhance high-temperature adaptability and ion conductivity; and the long carbon chain can strengthen the interaction between the xanthan gum and water molecules. With these advantages favorable for a high ionic conductivity, high salt durability, and high-temperature adaptability, xanthan gum shows a great potential to act as a HE. Zhang et al. presented xanthan gum electrolytes the adaptive and stable performances under high temperatures in RZIBs, including high capacities and rate capability, long-term cycling, and anti-deformation abilities [38]. Besides, xanthan gum acting as an electrolyte with 2 M ZnCl_2_ was reported by Kaltenbrunner et al. [39] to accelerate the ion migration. Due to the high salt tolerance, moisture retention, and ionic conductivity of xanthan gum HEs, the RZIBs showed a long lifespan and a high capacity. Note that after a 100% stretching, the xanthan gum electrolyte recovered in a short time and remained stable performances.

*Cellulose* Cellulose is macromolecular polysaccharide composed of glucose, thereby endowing intrinsic mechanical strength and flexibility so that it can serve as a electrolyte material to stable the properties of batteries. For example, carboxymethyl cellulose sodium (CMC) is a linear polymer composed of *β*-linked glucopyranose, which residues with carboxymethyl substitution. It has been widely used in HEs-based RZIBs because of its inherent biocompatibility, biodegradability, and nontoxicity. Cellulose, one of the most abundant natural polymers, can be easily processed into functional materials with enhanced properties through chemical or physical extracting and cross-linking [40]. Based on many works, it is believed that cellulose electrolytes possessed notable ion conductivity and chemical stability in a wide temperature range. Wang et al. [41] fabricated a conductive cellulose hydrogel (CCH) with high ionic conductivity and excellent anti-freezing properties. Specifically, cellulose was first dissolved in benzyltrimethyl ammonium hydroxide (BzMe_3_NOH) aqueous solution. After a chemical cross-linking process, this CCH was manufactured, which could directly serve as HEs. The anti-freezing properties of CCHEs resulted from uniform –OH groups in monomers. The profound mechanism was rich –OH groups generated a wider exothermic peak for the formation of ice crystals, possibly leading to the attraction between cellulose and water molecules, weakening the water-water interaction, and thus hindering the crystallization.

*Polyacrylamide (PAM or PAAm)* As one species of synthetic linear polymer with ordered molecules, polyacrylamide is self-adaptive because quantities of water-soluble amide groups (–CONH_2_) are beneficial to hydrophilicity and ion conductivity [42]. However, for pure PAM, it is difficult to obtain high mechanical properties. Chemical cross-linking is an effective approach to address this problem. A cross-linked PAM HEs with high mechanical property can be synthesized by using acrylamide (AM) monomer, initiator, and cross-linker. Strong covalent bonding could be revealed between groups of PAM polymer chains and cross-linker. The *N*, *N*′-methylenebisacrylamide is regarded as a perfect cross-linker when reacted with AM monomers, forming a strengthened and stable network structure in PAM. Zhi et al. used PAM as HEs in their works, which delivered great mechanical properties after cross-linking and favorable performance after assembling into RZIBs [43–45]. Zhu et al. [46] reported a similar cross-linking method of PAM HEs in their studies to support RZIBs to work in cold environments, whose specific process was mixing AM monomer, initiator, cross-linker, ZnSO_4_ salt, and an extra solution of 4 M LiCl as precursor solution and heating the solution to prepare PAM HEs. Thanks to the addition of 4 M LiCl, PAM HEs-based RZIBs could work at a freezing temperature of −20 °C.

*Polyvinyl Alcohol (PVA)* On basis of good self-healing ability, interface compatibility, and exceptional strength, PVA shows a prospect in RZIBs as a HE for energy flexible devices [47–49]. The PVA hydrogel shows a physical property with abundant hydroxy side groups and the O–H···O hydrogen bonds in chain segments. After cutting, PVA can independently recover to the original state (self-healing) without any extra assistance in the hydrogel matrix. Huang et al. fabricated the PVA/Zn trifluoromethanesulfonate (Zn(CF_3_SO_3_)_2_) HEs and measured their self-healing capability [50]. Results reflected the contacting again between hydrogen bonds after cutting to light the bulb. Besides, Li et al. introduced the carboxyl groups into main chain structures to further improve the recovery capability of PVA [51]. The carboxyl-modified PVA cross-linked by COO–Fe bonds as hydrogel network, Zn(NO_3_)_2_ and MnSO_4_ as electrolytes, the quasi-solid-state Zn–MnO_2_ batteries had better self-healing properties under serious cutting and punching.

#### Hybrid Species

Most of the hydrogels with a single component cannot meet the urgent demands for multi-functional electrolytes, particularly under harsh conditions [52]. Therefore, hybrid hydrogel electrolytes were designed by cross-linking two or more species of polymers, which generally exhibit multiple functions compared with the mono-component counterpart. For instance, Li et al. assembled PAM/gelatin hybrid HEs for quasi-solid-state RZIBs, which not only deliver high areal energy density, ion conductivity, and specific capacity but also show excellent mechanical strength, flexibility, wearability, and safety under the damages of cutting, bending, washing, compression, combustion, etc. [53]; Chen et al. developed a novel polyvinyl alcohol/nano-cellulose hybrid hydrogel via a borax-cross-linked method, where nano-cellulose could strongly interact with PVA chains by quantities of hydroxide groups and effectively enhanced the mechanical and self-healing properties of electrolyte to tolerate the bending and cutting damages [54]; and Mo et al. constructed EG-waPUA/PAM hybrid HEs, which can maintain stable performance after twisting many times under −20 °C [55].

However, it needs to be further discussed how to design and select monomer species and what is the matching principle between the different species in hybrid HEs. Generally, hybrid HEs have two kinds of inner structures: (1) single-network structure; (2) dual-network/multi-network structure. For the first kind, the matching monomer species need to have the same or similar chemical bonds to cross-link, such as –C=C–, –C≡C–, –COOH, –NH_2_, etc. The design and selection principles depend on the chemical groups, relative molecular weight, stoichiometric ratio, and solubility of each component. For the second kind, the inner structures of HEs are comprised of two or more networks, where each network forms by the self-polymerization of the same monomer. These networks display only a physical entanglement in HEs. It should be noted that the matched monomer species in this kind of HEs cannot be cross-linked/reacted with each other; otherwise, the multi-networks cannot be maintained.

### Development History of Hydrogel Electrolytes in RZIBs

In previous studies, hydrogel or gel polymer materials were frequently applied in LIBs and SIBs, which were usually realized by attaching flexible electrode materials with thin separators infiltrated by liquid electrolytes in between. However, this design displayed a neglected weakness in that flexible hydrogel would be apart from current collectors when mechanical deformations occurred. To overcome this problem, gel polymers directly served as both the electrolytes and the separators and offered protective effects to LIBs in 2011, with good ionic conductivity [35]. It was the first time that gel polymer was reported as an electrolyte in the batteries. In 2013, Chen et al. proposed the concept of “quasi-solid-state electrolyte” in rechargeable LIBs by fabricating a poly(methacrylate) (PMA)/poly(ethylene glycol) (PEG)-based gel polymer electrolyte (GPE) [56]. With the increasing demands for safety issues and the booming development of hydrogel materials, hydrogel polymer electrolyte (HPE) was derived from GPE. In 2018, Bae et al. reported a Li-ion electrolyte composed of cross-linked PVA-based 3D nanostructured hydrogel combined with Li_0.35_La_0.55_TiO_3_ (LLTO) frameworks for high-performance composites [57]. It was found that the as-fabricated HPE exhibited a high ceramic content of 44 wt% and an enhanced room temperature ionic conductivity of 8.8 × 10^−5^ S cm^−1^. In addition, Zhong et al. fabricated aqueous rechargeable SIBs by employing the PAM material as HPE in 2018, achieving a record-breaker ionic conductivity of 1.18 mS cm^−1^ at that time in this work [58]. It should note that hydrogel polymer electrolyte (HPE) is also abbreviated as HE, whose plural was HEs. Thus, all these concepts can be used to represent “hydrogel-based electrolytes” in the follow-up studies.

**Fig. 3:**
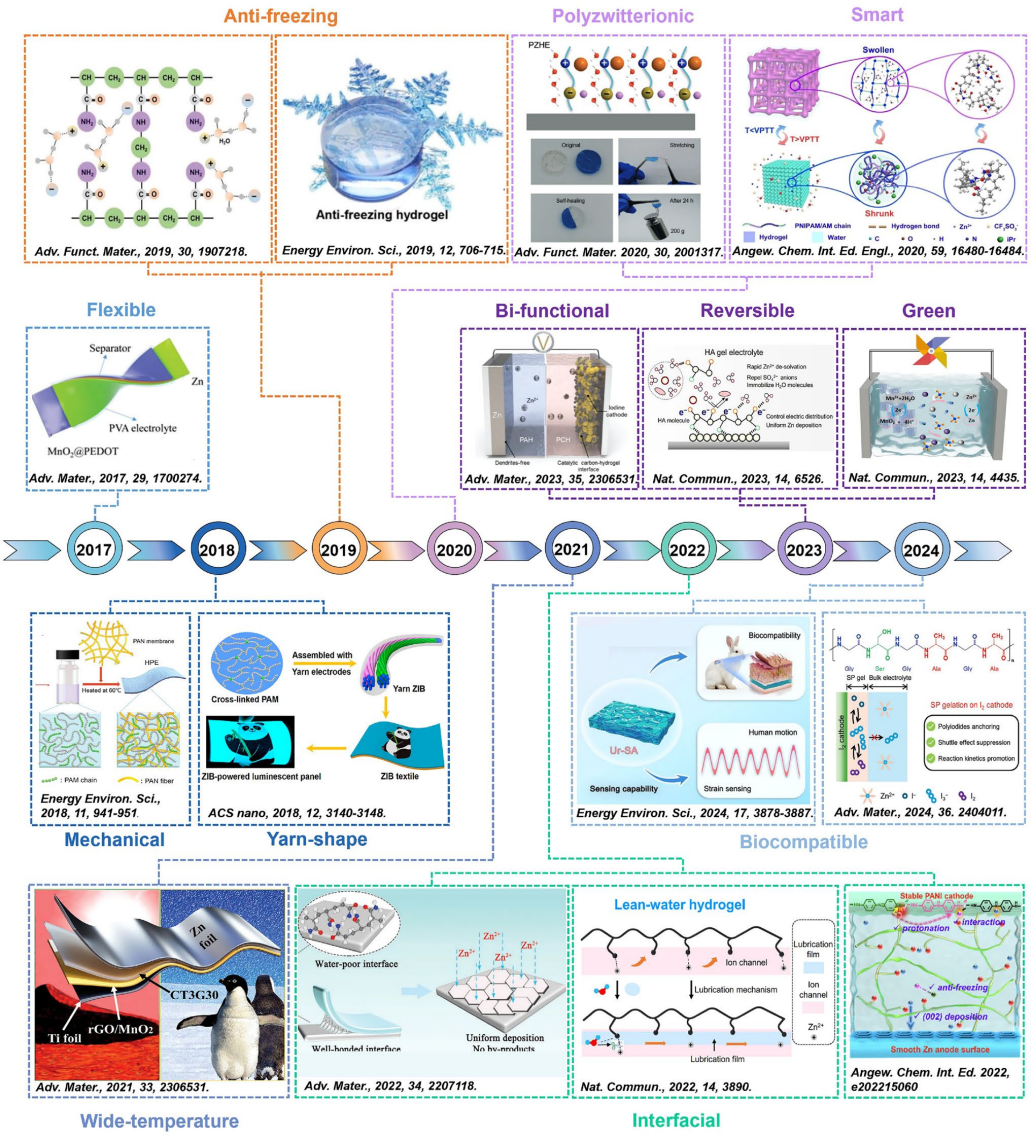
A brief development history of HEs-based RZIBs from 2017 to 2024

Besides the Li- or Na-ion energy storage techniques, the HEs also serve in rechargeable multivalent-ion energy storage techniques, such as Al-ion, Mg-ion, and Zn-ion batteries. Among these, HEs-based RZIBs have attracted more significant research interests, making safe and flexible energy storage to become another emerging field. Figure 3 presents the development history of HEs-based RZIBs to discuss the lots of works in this field. Lu et al. reported a flexible Zn–MnO_2_ battery using PVA/ZnCl_2_/MnSO_4_ hydrogel in early studies, with a maximum energy and power density of 504.9 Wh kg^−1^ and 8.6 kW kg^−1^, respectively [59]. They applied these PVA/ZnCl_2_/MnSO_4_ HEs into a Zn–MnO_2_ battery with one new kind of 3D porous cathode to reach a longer cycling lifespan. In 2018, Zhi et al. constructed one creative kind of hybrid hydrogel by fabricating PAM and gelatin as the special hierarchical polymer electrolytes (HPEs) in flexible Zn–MnO_2_ battery [53]. Results suggested that the as-prepared HPEs achieved a mechanical strength of 7.76 MPa (maximal stress) and an ionic conductivity of 1.76 × 10^−2^ S cm^−1^ at room temperature. The high safety after cutting, bending, hammering, puncturing, and sewing and the wearable functions of the assembled batteries were comprehensively described for the first time. This work demonstrated that the HEs were promising for the applications of wearable devices. Subsequently, they found anti-freezing HEs of EG-waPUA/PAM for Zn–MnO_2_ systems in another work to resist subzero temperatures in 2019 [55]. In the same year, Zhu et al. used LiCl/PAM as HEs, also confirming their anti-freezing performance [46]. After 2020, the development of HEs-based RZIBs was booming, and wearable electronics had a close connection with such energy storage devices. Zhang et al. designed flexible and high-voltage coaxial fiber RZIBs based on cellulose sodium [60]; Li et al. fabricated yarn-type RZIBs based on PAM [45]; and Niu et al. constructed smart HEs for the applications into flexible electronics [61]. In 2021, HEs developed toward multi-functional or multi-layer integration: Ni et al. summarized the multi-functional HEs under extreme conditions in their work [62]; Xia et al. comprehensively reviewed advances of HEs in aspects of their synthesis, characterization, performance validation, and interactions between electrodes and HEs [35]; Parkin et al. discussed the insights of functional HEs-based RZIBs from laboratory research to commercialization [25]. Some new and profound ideas were introduced into HEs for the better performance of RZIBs. Zhi et al. designed lean-water PAM hydrogel in 2022, which could quantify and orient water molecules in HEs [63]. Recently, the biocompatible hydrogel for Zn–Mn/V-oxides batteries and multi-functional hydrogel for Zn–I_2_ batteries have attracted researchers’ interests: Guo et al. proposed a biocompatible HEs by using hyaluronic acid [64]; Zhou et al. reported a biocompatible and flexible sodium alginate/urea composite hydrogel as the electrolyte [65]; Zhou and Xu et al. developed recyclable and biodegradable HEs based on natural biomaterials, namely chitosan and polyaspartic acid [66]; Fan et al. constructed a hetero-polyionic hydrogel as electrolyte, containing iodophilic polycationic hydrogel layer on the cathode side and polyanionic hydrogel layer toward Zn anode [67]; and Qiao et al. provided an interfacial gelation strategy to suppress the shuttle effects and improve the Zn reversibility simultaneously by introducing silk protein (SP) additive [68]. In conclusion, HEs-based RZIBs have prospered and many researchers will open a new era in this field.

### Interface Compatibility Between Hydrogel Electrolytes and Electrodes

**Fig. 4:**
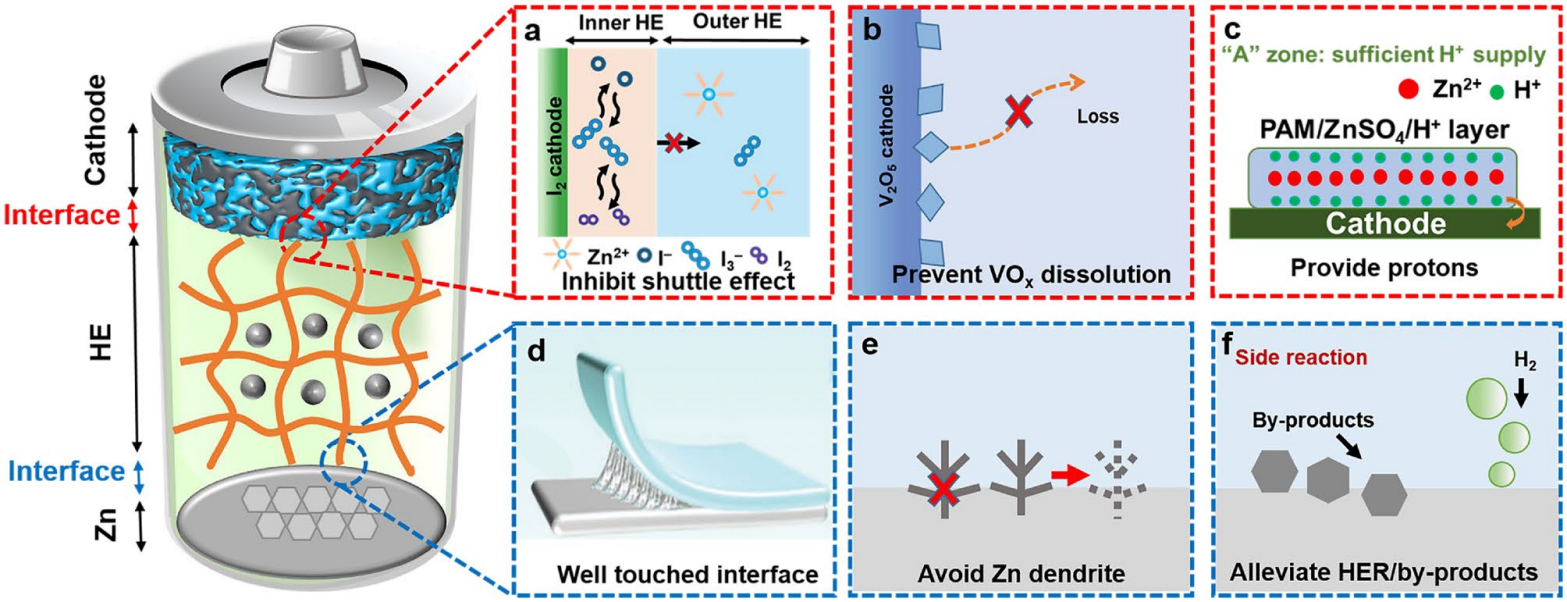
Effects of HEs on the interface of cathode/anode: **a** inhibit shuttle effect of polyiodides, **b** prevent the dissolution of active materials, and **c** provide protons for cathode; **d** well-touched interface with Zn, **e** avoid Zn dendrites, and** f** alleviate HER and by-products on Zn anode

The interface compatibility between HEs and electrodes significantly impacts the electrochemical performance of batteries (Fig. 4), making it a key research focus in HEs-based RZIBs [69]. The interfaces between HEs and anode/cathode will produce solid electrolyte interfaces (SEI) during the electrochemical process, which has vital influences on cycling lifespan, rate performance, CE, and battery overpotential [70]. Moreover, the interfacial reaction dynamics will regulate the parameters of ion conductivity and the ion migration rate. The prerequisite of good compatibility for RZIBs is that HEs can fit well with the anode/cathode materials. However, due to the quasi-solid state, the HEs have some limitations to meet the demands of solid-state electrode materials. The quasi-solid/solid interface is the uppermost issue in terms of compatibility. As a result, the quasi-solid state of polymer structure and the anions/cations gathering in hydrogel will cause the high impedance of electrolytes, high concentration polarization, low CE, and low voltage efficiency.

The design of hydrogel interface compatibility should focus on two key aspects: (1) HEs–cathode interface and (2) HEs–anode interface. For the HEs–cathode interface, the design principle of hydrogel is mainly focused on how to achieve strong adhesion with cathode materials and how to alleviate the loss of cathode materials by inhibiting the shuttle effect, preventing the dissolution of VO_*x*_ and MnO_*x*_, etc. For the HEs–anode interface, the design parameters mainly include adhesion strength of interface, ion conductivity, low impedance, etc., to avoid Zn dendrite and suppress side reactions and by-products. The high ion conductivity, low impedance, and well-touched adhesion strength of hydrogel polymers can be realized by optimizing ion mobility, ion distribution, and ion concentration in network structure. These factors guarantee HEs to obtain comparatively decent electrochemical performance for RZIBs [29].

#### Interface Compatibility Between Hydrogel Electrolytes and Cathodes

The material viscosity and surface tension of quasi-solid hydrogels ensure effective contact at the HEs–cathode interfaces. Various cathode materials for RZIBs include: (1) manganese (Mn)-based oxides, (2) vanadium (V)-based oxides, (3) Prussian blue analogs, (4) I_2_ or iodine compounds, (5) Li- or Na-ion-intercalated materials, (6) organic materials, and (7) bifunctional air electrode materials. As a result, diverse HEs design strategies are tailored to the specific characteristics of different cathode materials.

For I_2_ cathodes, the HEs can avoid the dissolution of polyiodide, further inhibiting the shuttle effect of polyiodides (I_3_^−^ or I_5_^−^) [14, 67, 68]. The shuttle effect of water-soluble I^−^, I_3_^−^, and I_5_^−^ causes irreversible loss of active materials, resulting in fast capacity decay and low CE. In addition, the sluggish reaction kinetics due to poorly conductive I_2_ will generate high polarization and low iodine utilization. During the charging/discharging process of batteries, the direction of the electric field is switched periodically, which leads to the bidirectional migration of ions in HEs with positive charge toward I_2_ cathodes. Zhang et al. proposed an interfacial gelation strategy to suppress the shuttle effects and improve the Zn reversibility by introducing the hydrogel layer in electrolytes, which could migrate bidirectionally toward cathode interfaces driven by the periodically switched electric field direction during charging/discharging [68]. Besides, in terms of I_2_ cathodes, the interaction between polymer and polyiodides formed a gelatinous layer to avoid polyiodide dissolution. Yang et al. designed one kind of hetero-polyionic hydrogel as the electrolyte [67]. On the cathode side, the iodophilic polycationic hydrogel layer effectively alleviated the shuttle effect and facilitated the redox kinetics of iodine species.

For the cathodes of vanadium oxides, the HEs can effectively prevent the dissolution of active materials. As the solvent in electrolytes, H_2_O plays a vital role in the ion kinetics mechanism. However, the water molecules will accelerate the loss of active materials, such as V_2_O5, which is likely to convert to V^3+^ to dissolve into the liquid electrolytes. Reducing the free water content in the HEs can inhibit the dissolution of the cathode materials. Zhi et al. demonstrated that HEs could suppress the dissolution of cathode materials and the side reactions by designing lean-water HEs [63]. Chen et al. discussed the inhibition in the case of V_2_O_5_ by constructing a rapid Zn^2+^-conducting HE (R-ZSO) to break side-reaction loops and simultaneously achieve dense Zn deposition [37]. In this work, the R-ZSO were manufactured from xanthan gum and boron nitride. This HEs had a strong affinity with SO_4_^2−^, which accelerated the migration of Zn^2+^ and realized the dense deposition. Furthermore, R-ZSO could hinder the migration of VO_2_(OH)_2_^−^ and H_3_O^+^, demonstrating its storage ability of protons to assist the stable electrochemical reactions at the interface.

For the cathodes of manganese (Mn) oxides, the proton supply from HEs is one of the key effects on the performance of batteries. Many works have proposed the H^+^ intercalation views and demonstrated its significant function on aqueous Zn–MnO_2_ batteries. It is considerable that HEs can not only provide continuous protons for cathodes to convert MnO_2_ to Mn^3+^/Mn^2+^ but also suppress the dissolution of materials for manganese oxide cathodes. In our previous works [71, 72], we designed a tri-layer HE with multiple functions to realize precise proton distribution, inhibiting active materials dissolution, and increasing the specific capacity. On the cathode side, MnO_2_ combined with protons to have adequate conversion to Mn^2+^, achieving a more powerful interface adhesion and a maximum specific capacity of 516 mAh g^−1^ for Mn-based cathodes.

#### Interface Compatibility Between Hydrogel Electrolytes and Zn Anodes

Quasi-solid-state HEs generally feature good compatibility to Zn metal anodes, ensuring both well-touched interface and uniform Zn deposition [73, 74]. The issue of dendrite growth faced by Zn anode in common neutral/alkaline electrolytes can be effectively suppressed by HEs [75, 76]. The formation of Zn dendrites is generally attributed to non-uniform Zn^2+^ diffusion/deposition and uneven surface electric field distribution on the Zn surface. During Zn deposition, the protrusions would form a stronger electric field, which induces the preferential accumulation and deposition of Zn^2+^ on cusps, ultimately leading to the formation of Zn dendrites, known as the “tip effect.” However, the chain/network structures and functional groups of HEs can regulate the Zn^2+^ distribution and electric field on Zn, which significantly guide smooth and uniform Zn electrodeposition. For example, Yang et al. developed cation-conduction dominated HEs to holistically enhance the stability of Zn anode [14]. Central to this design are the functional groups of the polymers chain. The covalently bonded –SO_3_^−^ groups on hydrogel chains maintain the concentration of anions near the anode surface throughout the Zn plating/stripping process, which contributes to a flat and dense Zn anode surface without Zn dendrites. Moreover, the transference number of major mobile Zn^2+^ in the hydrogel reaches a high value of 0.81.

In addition, HEs can inhibit the hydrogen evolution and by-product formation on Zn anodes. In thermodynamics, the electrochemical stability window of water is restricted to 1.23 V, which indicates easy water splitting and limits the output operating voltage. The H_2_ evolution will increase the local pH value near Zn (2H_2_O + 2e^−^ → H_2_ + 2OH^−^; Zn → Zn^2+^ + 2e^−^), thus triggering the by-product formation (e.g., 4Zn^2+^ + SO_4_^2−^ + 6OH^−^ + *x*H_2_O = Zn_4_(OH)_6_SO_4_·*x*H_2_O). Moreover, the H_2_ evolution will elevate the internal pressure of battery and subsequently lead to battery explosions and electrolyte leakage. However, water molecules are bound around the hydrophilic groups of the polymers in HEs, promoting the desolvation of Zn^2+^ and reducing the electrochemical activity. Then, the side reactions on the Zn anode are suppressed in HEs. For example, Zhi et al. developed a lean-water HE functionalized with hydrophilic and zincophilic sulfonate groups, which significantly broadens the electrochemical stability window and improves the reversibility of Zn plating/stripping [63].

Interface incompatibility arises from the mismatched characteristics between electrodes and electrolytes, especially under harsh conditions, leading to issues like electrolyte–electrode separation during serious deformations and reduced ionic conductivity at low temperatures. Although various strategies have been proposed to optimize the interface, these problems persist. Designing HEs to overcome these challenges under harsh conditions remains a critical area for future investigation.

## Design Strategies Toward Harsh Conditions

Batteries face severe challenges in daily applications due to unexpected circumstances, such as water immersion, sudden fires, extreme temperatures/climates, or mechanical distortions. These conditions can significantly impact battery performance and safety. Conventional aqueous RZIBs are more prone to degradation under harsh environments, whereas HEs-based RZIBs exhibit superior properties, such as enhanced thermal stability and mechanical robustness, to withstand such environmental emergencies. Therefore, it is critical to analyze the specific mechanisms of HEs in RZIBs and explore how to design these HEs to resist harsh conditions effectively.

### Harsh Temperatures

**Fig. 5:**
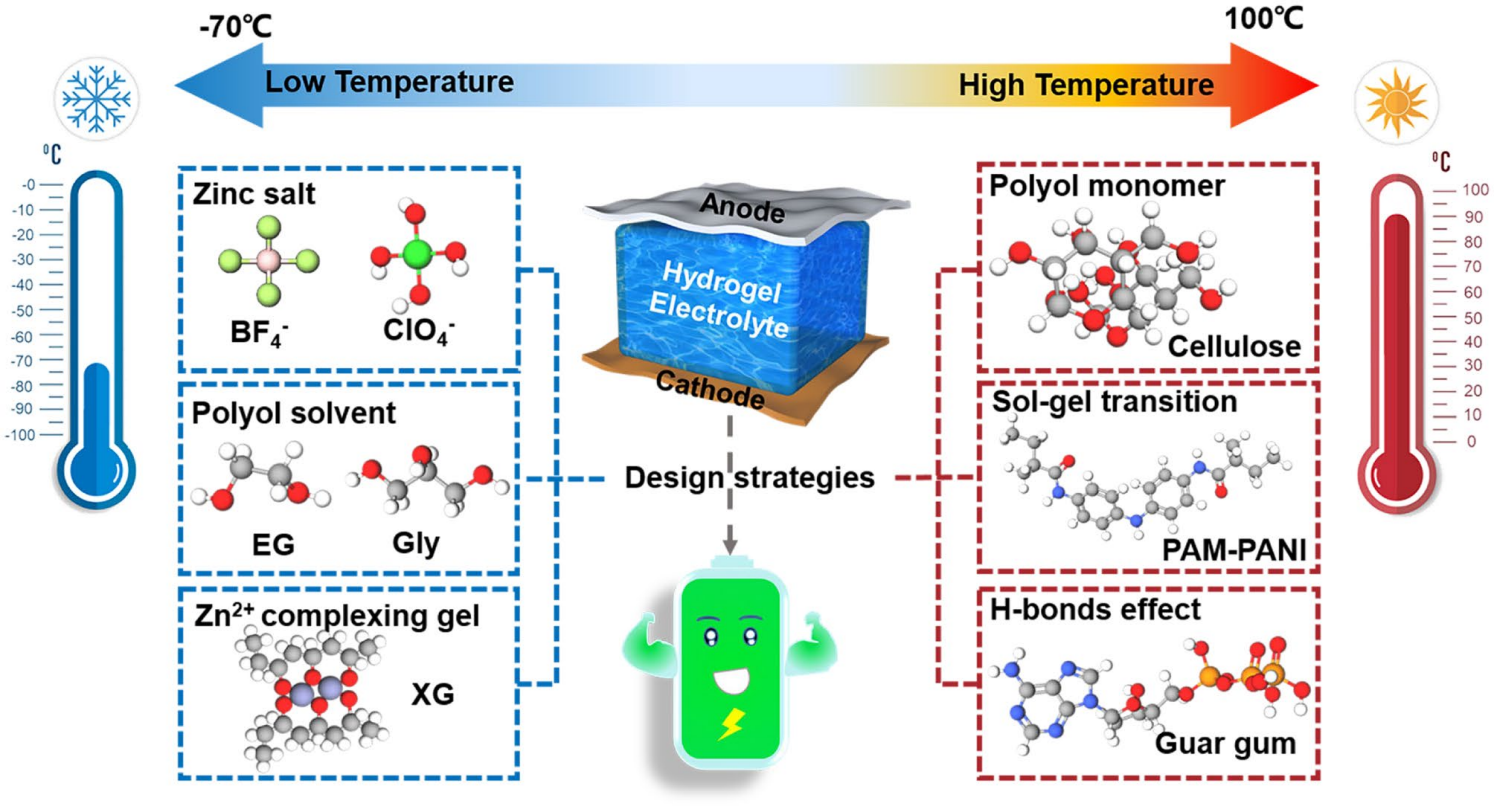
Design strategies for HEs-based RZIBs under low and high temperatures

Globally, temperatures may reach 50 °C in summer or drop below −40 °C in winter in many regions, reflecting the extreme impacts of climate change. For battery applications, it is essential to optimize the adaptability and performance of electrolytes under harsh temperatures, such as those exceeding 50 °C or subzero conditions, to ensure reliable operation and extended lifespan. Majority of HEs-based RZIBs are reported to work in suitable temperatures. With the increasing demands, the batteries need to normally operate under high/low temperatures or a wide temperature range [77]. However, conventional HEs-based RZIBs may not adapt to severe torridity or cold derived from harsh conditions. As a result, it will be a tremendous challenge how to design HEs to withstand extreme temperatures. For high temperatures, preventing dehydration and thermal shock is one of the main challenges of HEs design. For low temperatures, overcoming solidification by high entropy of H-bonds is one of the main challenges of HEs design. Practical strategies for addressing these issues resulting from harsh temperatures are summarized and evaluated (Fig. 5). Meanwhile, the recent progress of HEs-based RZIBs is introduced to explain these design strategies of HEs [78].

Inhibiting dehydration and thermal shock is the core idea of HEs design under high temperatures. Water evaporation occurs when the temperature exceeds 70 °C. During electrochemical reactions process, water splitting occurs when the voltage approaches or exceeds electrochemical window. So high-temperature batteries further accelerate the water loss in HEs, which directly lowers the ionic conductivity and rapidly fades the battery capacity [79]. The primary challenge for high-temperature batteries is preventing water evaporation/splitting and retaining moisture in hydrogels. An effective strategy is provided here that rich –OH groups can trap water in polymer structure of hydrogel. Typically, natural guar gum and xanthan gum possess the intrinsic thermostability due to abundant –OH, which can trap water in hydrogel and prevent water from evaporating at high temperatures. On the basis of rich –OH design, it is better to widen electrochemical window of HEs-based RZIBs to simultaneously suppress the water evaporation and splitting under high temperatures.

Preventing freezing is the pivotal idea of HEs design under low temperatures. In cold environments, battery performance deteriorates significantly due to reduced ion activity and increased internal resistance after freezing, leading to lower energy output and shorter lifespan [80]. This issue of poor performance in cold environments will be more pronounced in quasi-solid-state HEs, further limiting their practical applications. To overcome this problem, HEs need to be designed by introducing salt solutions of Zn(BF_4_)_2_, Zn(ClO_4_)_2_, LiCl, etc., such as Niu et al. introduced Zn(BF_4_)_2_ in PAM hydrogel, contributing to the stability of RZIBs under −70 °C [81]; adding polyol solvents of ethylene glycol, glycerol, etc., for instance, Liu et al. introduced glycerol into HEs to anti-freeze at −50 °C [82]; or modifying the hydrogel network by hydrophilic groups, e.g., Zhi et al. presented a novel waterborne anionic polyurethane acrylate/PAM (EG-waPUA/PAM) hydrogel system by introducing a new hydrophilic group hydroxyl and firmly anchoring it onto the hydrogel networks through covalent bond [55].

#### High Temperature

Many hydrogels are sensitive to high external temperatures due to water splitting or water evaporation [35]. These phenomena can significantly impact their performance and stability. Moreover, high temperatures adversely affect the structure and mechanical strength of hydrogel networks. Certain thermally cross-linked hydrogel polymers undergo structural changes at high temperatures, particularly those exhibiting sol–gel transition properties. To address these challenges, HEs-based RZIBs have been designed for enhanced heat tolerance, focusing on improving thermal stability and mechanical integrity under extreme conditions [79].

**Fig. 6:**
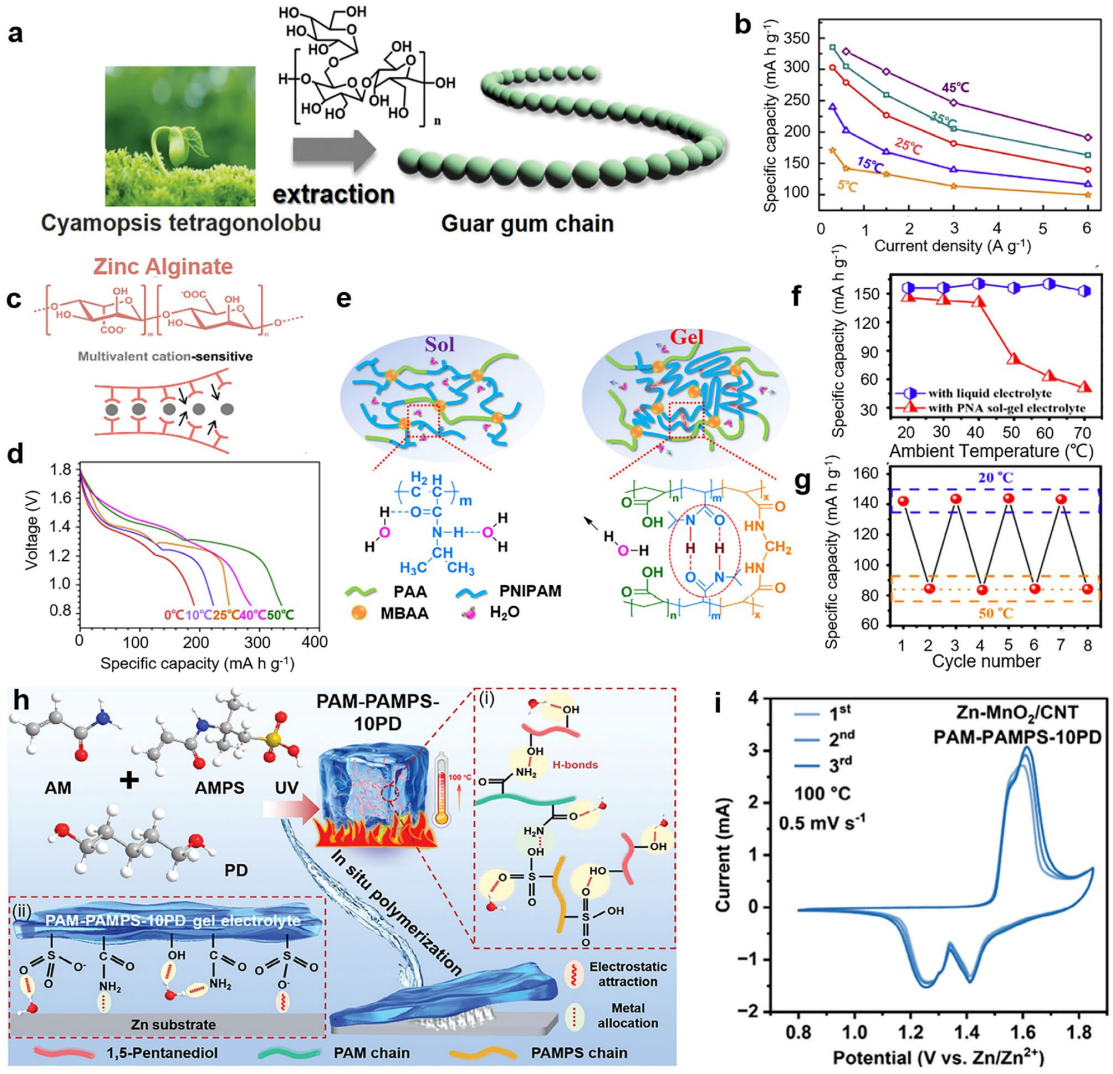
**a** Structure of guar gum. Reproduced with permission from Ref. [34]. Copyright 2022, Royal Society of Chemistry. **b** Rate performance of quasi-solid-state RZIBs with guar gum electrolytes at different temperatures. Reproduced with permission from Ref. [83]. Copyright 2019, Elsevier. **c** Zn^2+^-Alginate HE. Reproduced with permission from Ref. [84]. Copyright 2022, Wiley–VCH. **d** Discharging profiles of batteries with Zn alginate under different temperatures cycled at 0.2 A g^−1^. Reproduced with permission from Ref. [85]. Copyright 2020, Elsevier. **e** Synthesis of reversible poly (N-isopropylacrylamide) (PNI-PAM) sol–gel electrolyte. Reproduced with permission from Ref. [86]. Copyright 2018, Elsevier. **f, g** Specific capacities of Zn/*α*-MnO_2_ batteries with PNA sol–gel electrolytes calculated from discharge curves under various temperatures. Reproduced with permission from Ref. [87]. Copyright 2018, Elsevier. **h** Schematic design of a dual-network polyanionic PAM-PAMPS-10PD hydrogel when serving as electrolyte in Zn–MnO_2_ battery and **i** CV curves of battery at 100 °C. Reproduced with permission from Ref. [79]. Copyright 2022, Wiley–VCH

Due to their intrinsic thermostability, natural hydrogel polymers such as guar gum, xanthan gum, sodium/Zn alginate, and cellulose are widely utilized in designing HE network structures, providing enhanced thermal stability and mechanical strength. Additionally, HEs are engineered in sol–gel states to enhance their resistance to high temperatures, including PAM-PANI and PNA. For example, guar gum, a natural polymer extracted from Cyamopsis tetragonolobus (Fig. 6a), exhibits excellent thermal stability due to its robust molecular structure, enabling it to withstand high temperatures effectively [34]. Huang et al. prepared flexible and stable RZIBs with the conductive guar gum electrolytes in Fig. 6b, coupling *α*-MnO_2_-based hybrid cathode with carbon cloth current collectors and an electroplated Zn anode [83]. The discharging profiles and rate performances at 5.0 A g^−1^ are described at a temperature range of 5 °C ≤ T ≤ 45 °C. Such batteries deliver a specific capacity of 144.0, 176.4, and 193.9 mAh g^−1^ at room temperature (25 °C), 35 and 45 °C, respectively, demonstrating the stability of guar gum under higher temperatures. For rate performances, the batteries present the rising rate capability at different currents of 0.6, 1.5, 3.0, and 6.0 A g^−1^. Taking the current of 0.6 A g^−1^ as an example, the batteries showed a series of discharge capacities of 148.9, 212.3, 271.7, 308.6, and 328.5 mAh g^−1^ from 5 to 45 °C. In addition, Zn alginate (Alg–Zn) is another natural polymer with excellent thermal stability, making it suitable for applications in high-temperature environments [84, 85]. Due to the interaction between natural polysaccharide-sodium alginate and the active Zn^2+^, the cross-linked Alg–Zn HEs (Fig. 6c, d) were prepared via a direct ion cross-linking method with high-temperature tolerance. In Fig. 6d, it illustrated the excellent performance of Alg–Zn-based batteries within a temperature range of 0 °C ≤ T ≤ 50 °C, from 192.3 to 339.7 mAh g^−1^, and demonstrated a good adaptability of Alg–Zn HEs at a wider temperature range.

Additionally, an innovative design strategy for functional HEs is proposed here to enhance their high-temperature resistance, focusing on optimizing material composition and structural integrity. The hydrogel polymers are engineered as thermo-responsive HEs, enabling adaptive behavior under high-temperature conditions, and widely using in batteries, drug delivery systems, flexible materials, and wearable devices. Based on their superior thermal responsibility, HEs can present good performances under torridity. Mo et al. designed sol–gel HEs in Zn–MnO_2_ batteries (Fig. 6e): They synthesized *α*-MnO_2_/CNT nanocomposites cathode via a hydrothermal-coprecipitation method, coupling with Zn foil anode, and a reversible sol–gel transition electrolyte composed of poly(*N*-isopropylacrylamide-co-acrylic acid) (PNA) copolymer, which could smoothly work under high temperatures [86]. Figure 6f, g explains the reason that sol–gel HEs are conducive to maintaining the performance of batteries even at 70 °C [87]. The results of electrochemical cycling performance suggest PNA hydrogel lead batteries to a high and stable capacity over 142 mAh g^−1^ by thermal-dynamic transition at high temperatures. So the thermo-responsive sol–gel HEs endow the Zn batteries with significant charge/discharge rate performance under high-temperature conditions, which demonstrates an effective design for the thermostable batteries. Recently, Hou et al. developed a dual-network polyanionic HEs (denoted as PAM-PAMPS-10PD), which was capable of withstanding high temperatures (100 °C) by thermal-stimulus responsive design and in situ polymerization [79]. The abundant anionic groups in the electrolytes greatly improved Zn^2+^ transport, induced uniform deposition of Zn^2+^, and inhibited water activity by enhancing hydrogen bonding with H_2_O and changing the solvation structure of Zn^2+^ to alleviate Zn anode corrosion. As a result, the symmetric batteries showed stable cycling performance for at least 500 h at 100 °C and 0.5 mA cm^−2^/0.5 mAh cm^−2^ and realized dendrite-free Zn anodes at this temperature. Moreover, the full batteries have a capacity retention of 47.8% after 3000 cycles at 100 °C and 4 mA cm^−2^. This strategy leverages H-bond interactions between PAM and PAMPS/PD. The introduction of the H-bonds crowding agent PD into HEs increase the operating temperature range (100 °C), and the synthesized hydrogel possesses high ionic conductivity and high-temperature tolerance because of a large number of H-bonds. The mechanism is that on one hand, the abundant anionic groups in the designed electrolytes have strong interactions with Zn^2+^, which can induce the mobility of Zn^2+^ under high temperatures; on the other hand, the interaction between molecular crowding agent PD and water molecules reduce the activity of water molecules, thus alleviating the HER and side reactions, and increasing the boiling point of PAM-PAMPS-10PD gel electrolytes to achieve high-temperature adaptability and stability.

Hence, two design approaches are proposed for HEs-based RZIBs operating at high temperatures to enhance performance and stability. The one is to choose the natural hydrogels as precursors, i.e., guar gum, sodium alginate (Alg–Na), gelatin, which can sustain stable ability under high temperatures due to the intrinsic properties. These species are essentially polyhydroxy or high-boiling-points compounds as the polymer networks of HEs, with no or little water loss at high temperatures. The other idea is to design thermal-sensitive HEs by using specific compounds containing highly active chemical components as a precursor. Uniformly mix raw materials in the liquid phase with subsequent chemical hydrolysis and condensation reactions. The sol process is prepared by aging colloidal slow polymerization, while the gel process is fabricated by forming a three-dimensional network structure of hydrogel. Such HEs can be converted to liquid electrolytes under high temperatures and spontaneously revert to a quasi-solid state after cooling down. Based on two strategies, the desired HEs-based RZIBs can be achieved to normally work under high temperatures.

#### Low Temperature

The anti-freezing design of HEs in batteries is a promising area for addressing harsh temperature conditions, as it ensures stable ion transport and prevents electrolyte freezing at subzero temperatures [54]. Numerous studies have been developed HEs to operate effectively at subzero temperatures [88]. There are three anti-freezing strategies for HEs: (1) adding polyol solvents, such as ethylene glycol, glycerol, etc.; (2) introducing salt solutions, such as Zn(BF_4_)_2_, Zn(ClO_4_)_2_, and LiCl; and (3) modifying the hydrogel network structures.

**Fig. 7:**
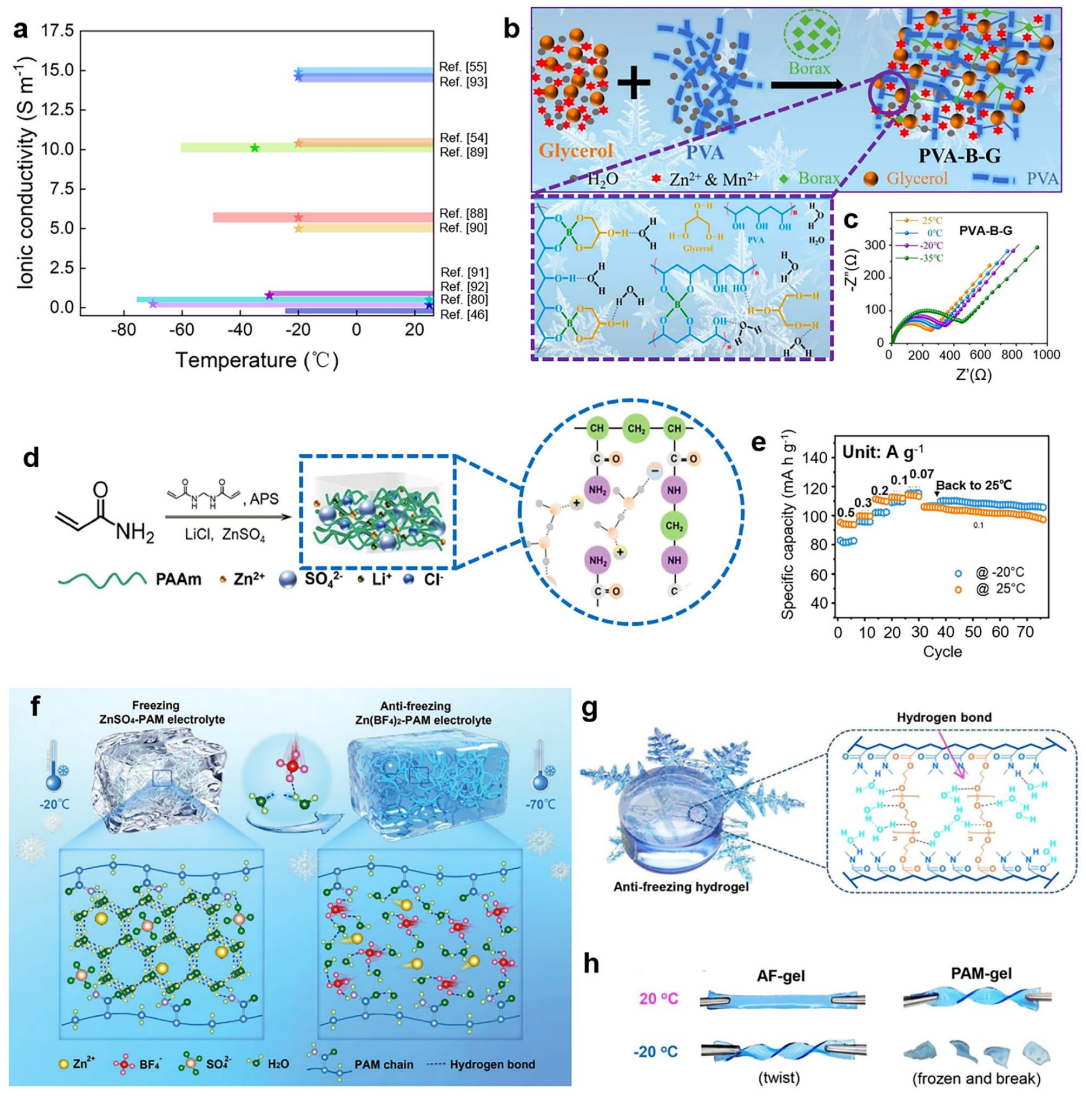
**a** A summarized chart for the ionic conductivity of reported HEs under low temperatures. **b, c** Designed mechanism and EIS spectra of hybrid PVA-Borax-Glycerol hydrogel in RZIBs under the temperature range from −35 to 25 °C. Reproduced with permission from Ref. [54]. Copyright 2020, Royal Society of Chemistry. **d, e** PAM hydrogel with LiCl additive as electrolytes in RZIBs and its electrochemical performance over the temperature range from −20 to 25 °C. Reproduced with permission from Ref. [46]. Copyright 2022, Royal Society of Chemistry. **f** Schematic diagram of PAM hydrogel under −70 °C. Reproduced with permission from Ref. [81]. Copyright 2023, Wiley–VCH. **g, h** Design principle of AF hydrogel and its good twisting performance under −20 °C. Reproduced with permission from Ref. [55]. Copyright 2019, Royal Society of Chemistry

We have collected lots of works about HEs-based RZIBs toward anti-freezing design in Fig. 7a [46, 54, 55, 81, 88–93]. Each work is displayed by using different color bars. The left side of the bar represents the lowest temperature batteries can withstand, and the star marks show the ionic conductivity values of HEs in RZIBs under the corresponding temperatures. From Fig. 7a, it is found that the lowest temperature HEs-based RZIBs can achieve is −70 °C among current works. To survive at subzero temperatures, batteries must be assembled by special HEs with a lower freezing point. We have mentioned three kinds of design strategies of HEs for RZIBs to resist low temperatures. The first anti-freezing strategy is to add polyol solvents. Chen et al. [54] fabricated a PVA HE by introducing Glycerol and explained its synthetic mechanism in Fig. 7b. Conventional PVA hydrogel is liable to fracture below −60 °C. However, the additive glycerol introduces abundant hydroxyls, which can combine with free H_2_O by hydrogen bonds. From free to the combined states, H_2_O molecules in HEs break the freezing point by enhancing the quantity of hydrogen bonds and effectively prohibits the ice crystals within the whole network [94]. Gathering with the Zn foil anode and rGO/MnO_2_ cathode, the anti-freezing PVA/glycerol are employed in Zn–MnO_2_ batteries, whose CV performances are examined at 1 mV s^−1^ in a temperature window of 25 to −35 °C. Figure 7c shows the electrochemical impedance spectroscopy (EIS) curves in this temperature range, illustrating the great anti-freezing performance of PVA/Glycerol HEs in batteries. The second anti-freezing strategy is to increase the solute concentration in electrolytes [95]. Decreasing freezing point by increasing the solute concentration is a common phenomenon in daily life, CaCl_2_ is widely applied to prevent roads from icing over, and anti-freezing seawater is also an example as the result of the mixture of salts in water. Zhu et al. successfully applied this approach in constructing their anti-freezing HEs [46]. They added cooperative Zn and lithium ions in hydrogel to enhance concentration of cations and dropped the freezing points, as depicted in Fig. 7d, e. Shi et al. presented a similar preparation method of HEs by adding Zn(BF_4_)_2_ concentration in their work (Fig. 7f), which conduce to the operation of HEs-based batteries at the lower temperature of −70 °C [81]. The third anti-freezing strategy is to optimize the structure of hydrogel to achieve a low freezing point. It was found that Mo et al. fabricated the hybrid anti-freezing hydrogel in a water/organic system by the combination of oleophilic and hydrophilic heteronetworks in Fig. 7g [55]. At −20 °C, the prepared hydrogel still maintained flexibility under violent twisting in Fig. 7h.

#### Wide Temperature Range

In practical applications, batteries urgently need to operate reliably under extreme temperatures. However, there is a growing demand for batteries to function across a wider temperature range. To achieve this, designing HEs with enhanced adaptability is critical. Most reported strategies for wide-temperature operation address the challenges of electrolyte condensation and partially mitigate water splitting in HEs by increasing the boiling point or reducing the water content in the electrolytes. As a result, RZIBs capable of wide-temperature tolerance generally exhibit performance at both high and low temperatures. However, adapting to a wide temperature range requires more complex construction or fabrication of hydrogel structures, often involving advanced material engineering and precise control over network properties.

**Fig. 8:**
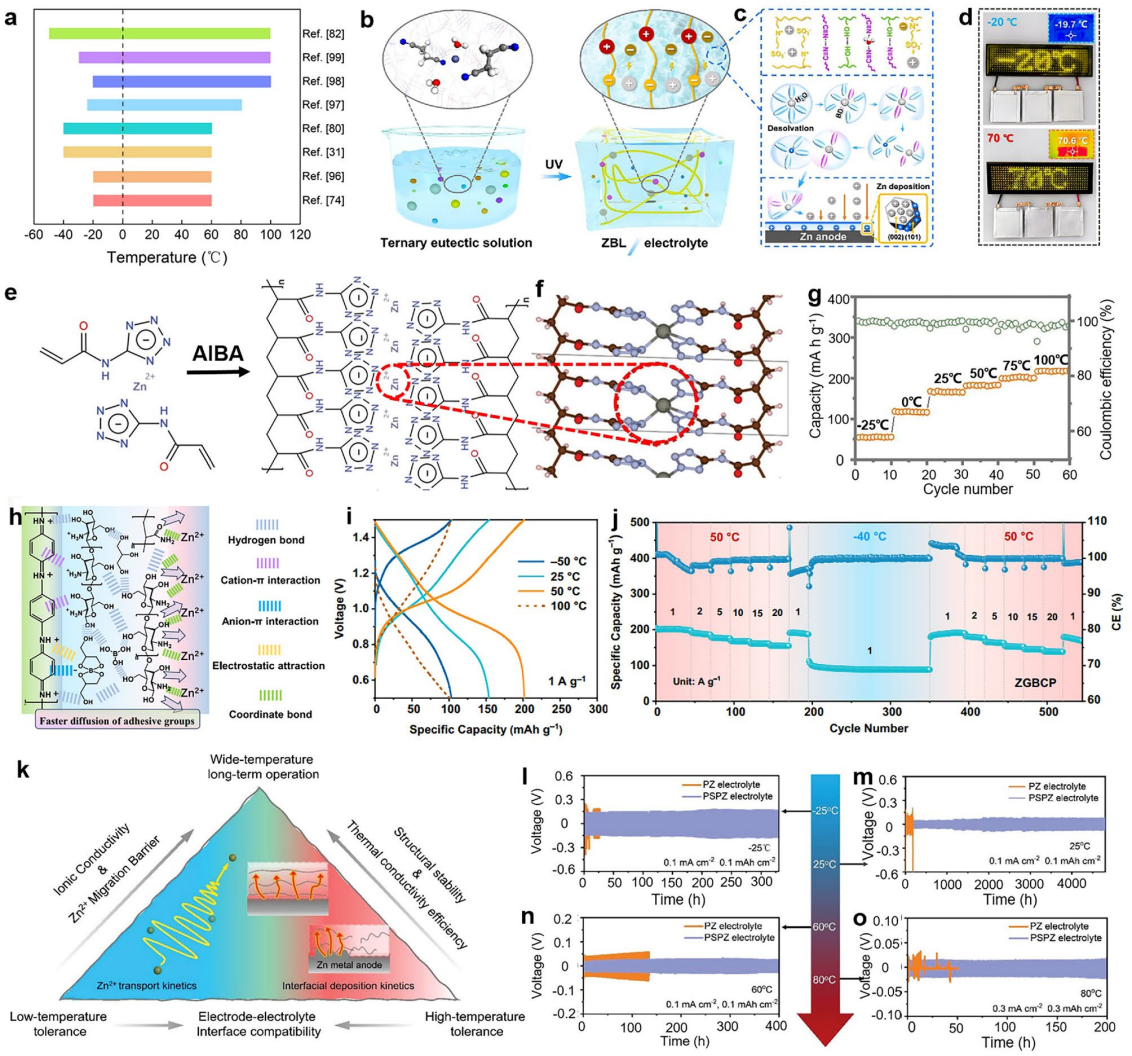
**a** A summarized chart of reported HEs with different operating temperatures. **b-d** Design mechanism and performance of HEs in RZIBs under a wide temperature range of −20 to 70 °C. Reproduced with permission from Ref. [100]. Copyright 2024, Wiley–VCH. **e** Polymeric (PSIC)-based HEs, **f** molecular structure, and **g** rate performance from −25 to 100 °C. Reproduced with permission from Ref. [99]. Copyright 2022, Wiley–VCH. **h** Schematic illustration of the adhesive mechanism of ZGBCP HEs, comprising of boric acid, glycerol, chitosan, and PAM, **i** the charging/discharging profiles under a wide temperature range from −50 to 100 °C at 1 A g^−1^, **j** and cycling performance of RZIBs with ZGBCP electrolytes at −40/50 °C. Reproduced with permission from Ref. [82]. Copyright 2024, Nature Publishing Group. **k** The design route toward wide-temperature HEs of PAN/SiO_2_/polyethylene oxide/Zn(OTf)_2_ (PSPZ) and the different electrochemical performance of assembled Zn/Zn half cells at temperatures of **l** −25 °C, **m** 25 °C, **n** 60 °C, and **o** 80 °C. Reproduced with permission from Ref. [97]. Copyright 2023, Wiley–VCH

We have tidied lots of works about HEs-based RZIBs toward wide-temperature design and selected some representatives to show in Fig. 8a [31, 74, 80, 82, 96–99]. Each work selected is marked by a unique color bar with the quoted reference. The left side of the bar reveals the lowest temperature batteries can normally work, while the right side of the bar indicates the highest temperature. Recent studies demonstrate that HEs-based RZIBs can operate over a temperature range from −50 to 100 °C, which basically covers the range of global climate conditions. Although this range of temperature is quite wide, the related records are constantly being broken as time goes on to explore the capable limit of HEs-based RZIBs toward adaptable temperature range. As mentioned, the HEs must possess both high and low-temperature stability in a wide temperature range. So the wide-temperature HEs must be designed toward both high-temperature strategies and low-temperature strategies.

Qiu et al. introduced acetamide molecules in electrolytes, which could disrupt the original H-bonded network of water molecules, replace the H_2_O in Zn^2+^-solvation sheath, form dynamic adsorption on Zn anode, and create an H_2_O-poor electrical double layer [74]. Moreover, the acetamide probably combined with Zn^2+^ to convert to larger complex molecules. Results suggested this strategy achieved a wide-temperature work range of −20 to 60 °C for RZIBs. Yan et al. designed a ternary deep eutectic solvent-based flexible HEs to address the adapting problems of wide temperature ranges of RZIBs [100]. Comprised of ZnClO_4_·6H_2_O, butanedinitrile (BD), and LiCl, the amphoteric hydrogel matrix enabled RZIBs to work at a wide temperature range from −20 to 70 °C (Fig. 8b–d). The lifespan of batteries using hydrogel matrix increased at high temperatures due to improving of interfacial stability and suppressing of side reactions. Except for organics addition, Chen et al. reconstructed hydrogel networks by using PSIC raw materials, which originally reduced the effect of water in HEs (Fig. 8e) [99]. Moreover, such a reconstructed structure could bound Zn^2+^, form Zn^2+^ transferring channel, and further increase ion conductivities of electrolytes (Fig. 8f). Rate performance of capacity indicated that the assembled batteries could stably work in a wide temperature range of −25 to 100 °C (Fig. 8g). The cotton was generally used in cold environments to insulate the heat and defend against the cold, so Chen et al. extracted cellulose from cotton as the raw materials of hydrogel [60]. As cross-linker, Si–O–Si–OH groups strengthened the heat/cold insulation of hydrogel (Fig. 8h). Rate performance of specific capacity (Fig. 8i) and corresponding galvanostatic charge–discharge (GCD) curves (Fig. 8j) demonstrated that CT3G30 HEs could operate from −40 to 60 °C. Li et al. directly described a wide-temperature design strategy of HEs in Fig. 8k, which pointed out many factors that needed to be considered, such as ion conductivity, ion transport kinetics, interfacial effect, and thermal conductivity. [97]. Based on this strategy, the prepared HEs could be adaptable to a range of −25 ~ 80 °C (Fig. 8l–o).

### Harsh Mechanical Deformations

Flexibility is a crucial parameter to evaluate the mechanical strength of HEs because of the important influence on the batteries performance. When facing to the extreme deformations, the hydrogel will tend to be cracked or damaged immediately. So robust mechanical stability of HEs will be urgently demanded for RZIBs to resist harsh deformations. Nevertheless, how to precisely evaluate flexibility is not defined. To date, the mechanical properties of hydrogels can be characterized through straining, stressing, bending, and even twisting tests, whereas the criterion of mechanical stability associated with electrochemical performance still needs to be implemented. Herein, the performance is conducted to discussions under four main deformations most likely occur.

#### Tensile Test

**Fig. 9:**
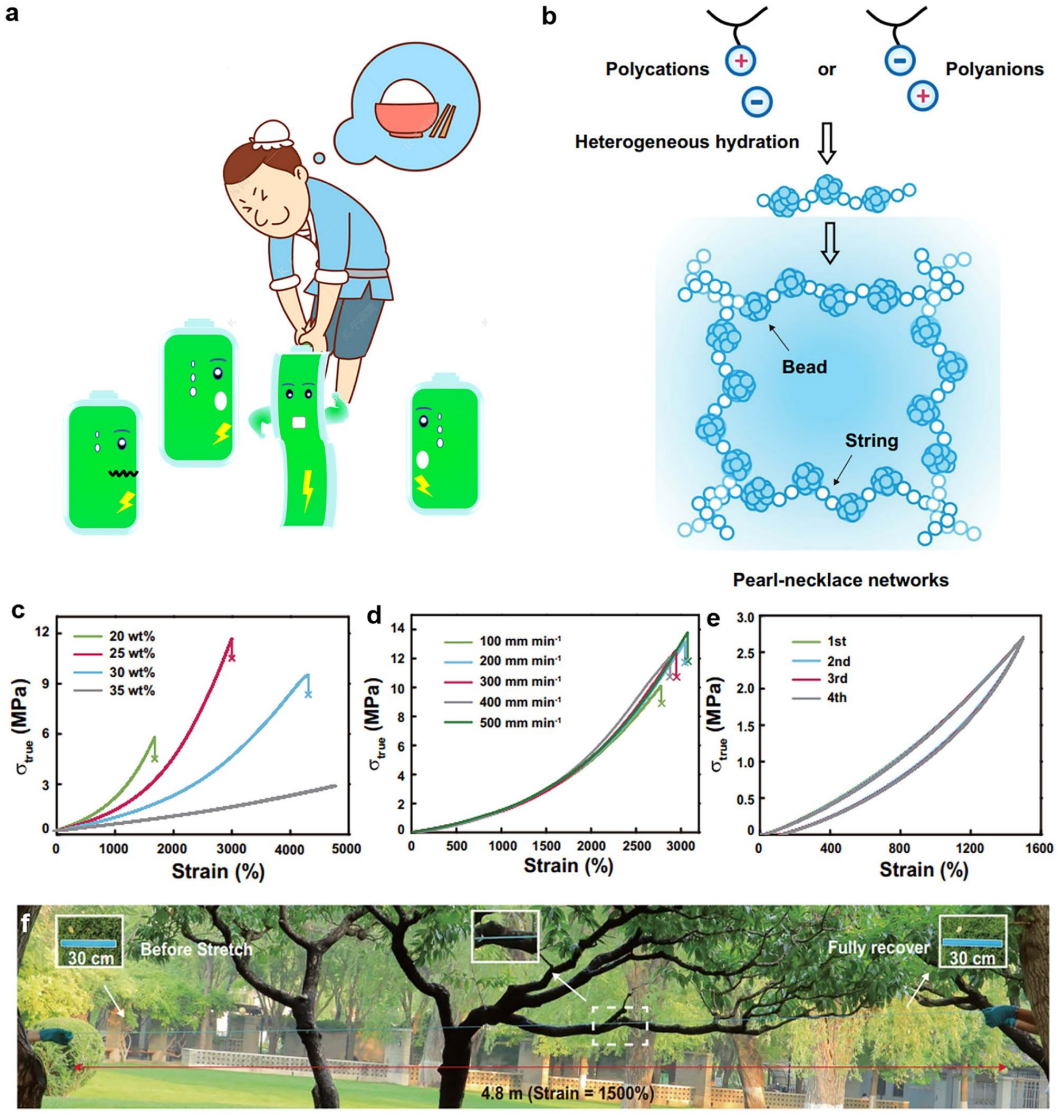
**a** A schematic diagram of the straining battery with HEs. **b** The pearl-necklace network of the hyperelastic hydrogel. **c** True stress–strain curves of the hydrogel with different water contents. **d** True stress–strain curves of the hydrogel at different loading rates. **e** Cyclic true stress–strain curves of the hydrogel at a strain of 1500% for four cycles. **f** A photograph of AETC-25 hydrogel (using poly[2-(acryloyloxy) ethyl] trimethylammonium chloride (PAETC) as building blocks) stretched to 1500% and recovering in seconds. The hydrogel section on the left shows the hydrogel before stretching. Reproduced with permission from Ref. [102]. Copyright 2024, American Association for the Advancement of Science

Straining, which measures the deformation response under stress, is one of the most critical parameters for evaluating the flexibility of HEs, as it directly reflects their ability to withstand mechanical deformation. Recently, more attention has been paid to ductile materials with great stretchability for the development of flexible and wearable devices-based HEs [101]. The elasticity is a very indispensable character for hydrogel straining as some polymer species tend to fail to recover their shape and size after releasing (Fig. 9a). By tensile test, the parameters of strain–stress and elastic modulus (also known as “Young’s modulus”) are obtained. The elastic modulus is calculated in the initial linear range from the stress–strain curves. The high elastic modulus of HEs should be pursued for preventing the deformation of straining. Figure 9a displays the straining states of HEs. For the quantification of straining parameters, stretchable rates of HEs are discussed in numerous studies. Earlier studies showed a photograph of stretched xanthan polymer with stretchable ratios of 25%, 50%, and 100%, presenting the mechanical robustness of hydrogel under the straining [39]. Some recent works [102, 103] reported more striking stretchable rates of 5 ~ 7 times and elastic modulus of 21 ~ 80 kPa. Unexpectedly, PAA and PAM HEs were confirmed the ultrahigh stretchable rates of 25 ~ 30 times by Zhang et al. and Li et al. [45, 104]. Figure 9b presents a hyperelastic hydrogel of PAETC. In this case, hydrogel can achieve an ultra-large areal straining ratio of 5000% ~ 10,000%, with an elastic modulus of 0.5 ~ 1 MPa (Fig. 9c–f).

Based on these studies, some feasible strategies are concluded to fabricate a more flexible hydrogel by strengthening the mechanical strength. Adding fillers is one of the most widely used methods to enhance the tension resistance of hydrogel. Two directions are provided here: (1) increasing the concentration of monomers; (2) selecting the suitable cross-linkers and inducers. Rare hydrogels have strong structures with large and regular molecules, except for some natural species, e.g., xanthan gum, cellulose, and sodium alginate. [63]. The monomers should be regular and sufficient in hydrogel to support a large and dense structure of network without obvious swelling. Besides, for the polymerization of hydrogel species, it is essential to select suitable cross-linkers and inducers to effectively fabricate polymer structures with great mechanical strength [56]. Typically, Zhang et al. introduced hydrogen bonds in cross-linkers into PAA hydrogel chains, strengthening the strain capacity of the initial PAA polymers [104].

#### Compression Test

Compression and straining are opposing types of deformation, yet both are fundamentally linked to the mechanical strength of hydrogels. Hydrogels, owing to their inherent flexibility and elasticity, can typically withstand varying degrees of compression. However, to endure significant compressive stress, HEs must exhibit high mechanical strength, which is essential for preserving structural integrity under high compression and ensuring reliable performance in real-world applications.

**Fig. 10:**
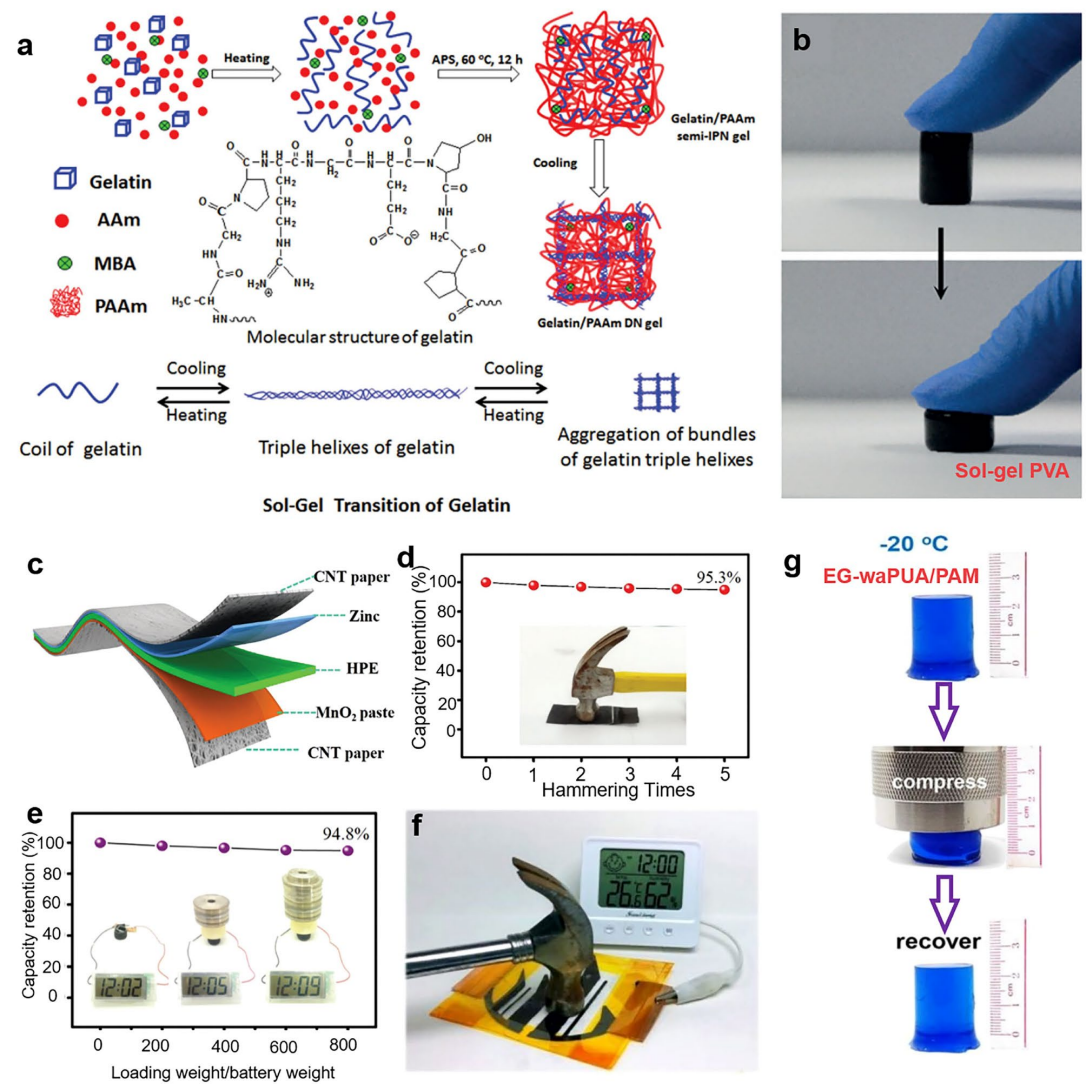
**a** Preparation process of PAM/gelatin hydrogel. Reproduced with permission from Ref. [105]. Copyright 2016, Royal Society of Chemistry. **b** Compression test of sol–gel PVA under thumb pressure. Reproduced with permission from Ref. [106]. Copyright 2016, Wiley–VCH. **c** Schematic illustration of the structure of the solid-state ZIBs, and its electrochemical performance in different destructive **d** Hammering test and **e** Weight loading test. Reproduced with permission from Ref. [53]. Copyright 2018, Royal Society of Chemistry. **f** Safety tests of Ur-SA-based planar RZIB under hammering. Reproduced with permission from Ref. [65]. Copyright 2024, Royal Society of Chemistry. **g** Elastic stability test of AF-gel for compression behaviors at −20 °C. Reproduced with permission from Ref. [44]. Copyright 2019, Royal Society of Chemistry

The PAM/gelatin composite hydrogel (Fig. 10a) is a typical example containing robust networks because of the interlaced combination of AM and gelatin monomers [105]. Under the action of ammonium persulfate (APS), the PAM and gelatin were interlinked as a stable polymer network by the dehydration synthesis reaction between amidogen and carboxyl from two respective precursors, which had high anti-compression ability in Fig. 10b [106]. Due to the high elasticity and attainable fabrication, the utilization of PAM/gelatin hydrogel in RZIBs has been frequently reported. Li et al. manufactured such hydrogel as electrolytes in a conventional way [53]. Integrating flexible MnO_2_ cathode and Zn anode, they assembled a novel RZIBs with superior flexibility in Fig. 10c. To detect whether the PAM/gelatin-based Zn–MnO_2_ batteries can withstand a sudden compression or impact, the consecutive hammering damages are simulated in Fig. 10c. It was found that the RZIBs could maintain an over 95% capacity retention after five violent hammer strikes, in which the highest impact of instantaneous pressure was estimated to be more than 3 MPa. The batteries based on PAM/gelatin were also found capable of loading heavy pressure in Fig. 10d, e, which indicated an excellent robustness of PAM/gelatin HEs under harsh deformations.

Besides, the sodium alginate/urea (Ur–SA) HEs were demonstrated a good loading ability under compression in Fig. 10f [65]. However, not all hydrogel species can be recovered to what it was to be, and some species suffer from the self-shrinkage after overload. Zhao et al. compared three species of hydrogel in their work, and only the cellulose electrolyte cross-linked by a synergetic chemical–physical approach, could recover its original shape, while the cellulose HEs cross-linking by single chemical or physical approach were immediately destroyed under large pressure (Fig. 10g) [44].

How can HEs be designed with mechanical stability under heavy compression? Two main strategies are proposed to enhance their mechanical performance. The first strategy focuses on optimizing the fabrication method of hydrogel polymers. For instance, Zhao et al. employed a hybrid approach combining chemical and physical cross-linking, rather than relying solely on one method, to significantly enhance the mechanical strength of cellulose [44]. The second strategy involves strengthening the network structure by incorporating suitable additives, such as new monomers, functional organics, and salts, which must be compatible with the main hydrogel structure. For example, Wang et al. designed CCH electrolytes by adding BzMe_3_NOH demonstrating excellent elasticity of HEs even after compression at −40 °C [41]. The incorporation of BzMe_3_NOH, which is rich in hydroxyl groups, not only improves mechanical stability but also imparts anti-freezing properties to the hydrogels by disrupting ice crystal formation and enhancing hydrogen bonding within the network. Similar additives with functional groups, such as hydroxyl-, disulfide-, and ammonia-based compounds, have also been explored for their ability to enhance hydrogel performance through mechanisms like dynamic bonding and improved interfacial interactions.

#### Bending Test

**Fig. 11:**
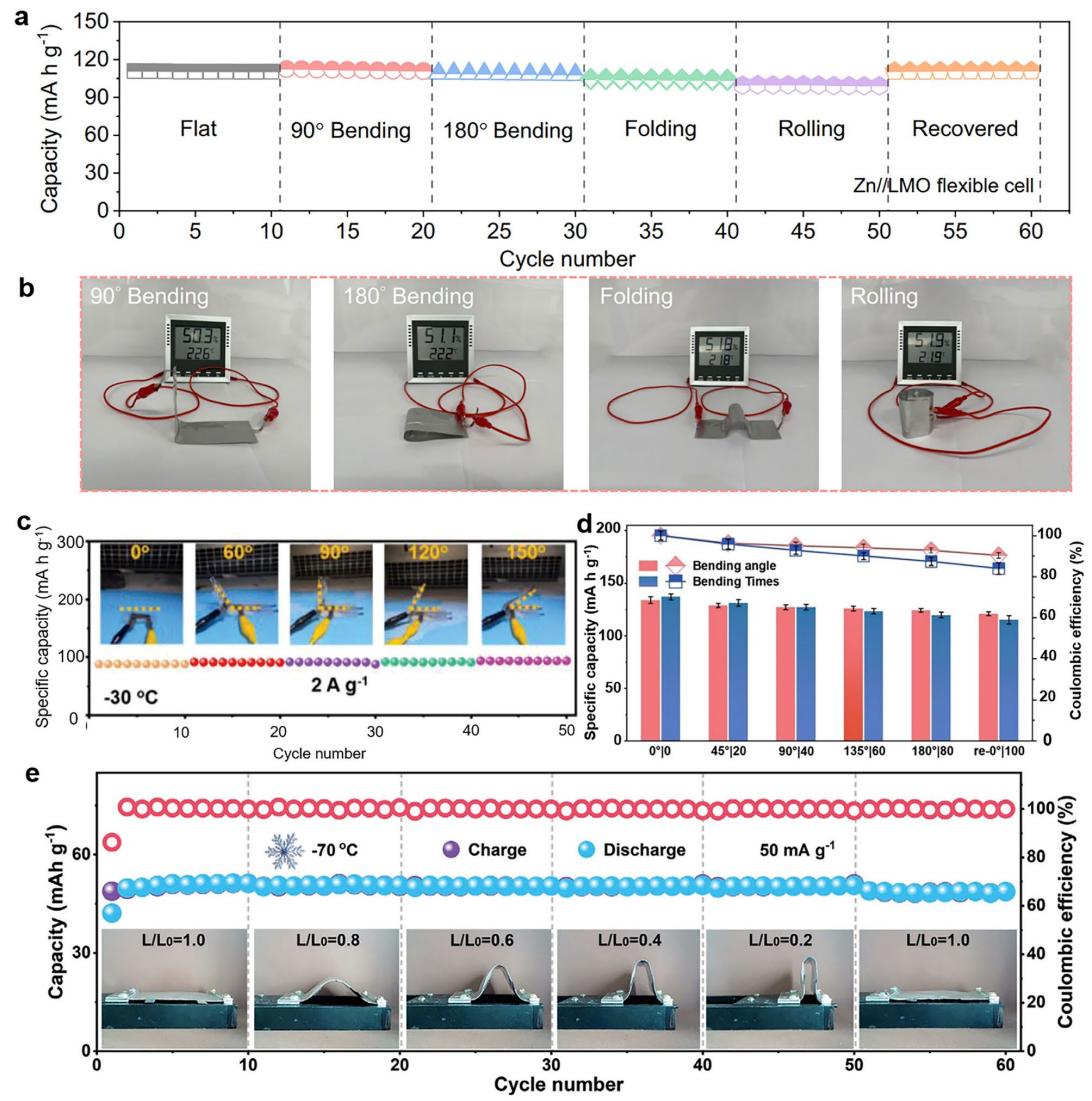
**a** Cycle performance of flexible RZIBs using HEs and **b** optical photographs of a hygrometer powered by the flexible RZIBs under varied mechanical deformation conditions. Reproduced with permission from Ref. [64]. Copyright 2023, Nature Publishing Group. **c** Practical demonstration of batteries under different bending angles at 2 A g^−1^ under −30 °C. Reproduced with permission from Ref. [79]. Copyright 2023, Nature Publishing Group. **d** Specific capacity changes at different bending angles from 0° to 180°. Reproduced with permission from Ref. [65]. Copyright 2024, Royal Society of Chemistry. **e** Cycling performance of anti-freezing flexible RZIBs at 50 mA g^−1^ under different bending states at −70 °C. Reproduced with permission from Ref. [81]. Copyright 2023, Wiley–VCH

As a critical method for evaluating flexibility, the bending test directly reflects the ability of HEs in batteries to withstand mechanical deformation during operation. Most research has emphasized the mechanical deformability of HEs-based RZIBs, with bending degree being a key parameter for wearable and flexible electronics applications. Thanks to their inherent elastic properties, nearly all HEs can be bent, but the degree of bending depends on factors such as composition, cross-linking density, and network structure. Li et al. constructed Zn–LiMn_2_O_4_ batteries with biocompatible HEs and investigated various mechanical bending performances (Fig. 11a, b) [64]. HEs-based RZIBs maintained high capacity retentions after both 90° and 180° bending tests, demonstrating the excellent properties of bending at room temperature.

However, some hydrogels cannot be bent freely to wide angles or multiple times, especially at subzero temperatures, due to their increased brittleness. For this case, Huang et al. fabricated polysaccharide HEs-based Zn/polyaniline (PANI) battery and found the assembled batteries could smoothly work at different bending angles under −30 °C [79]. Zhang et al. recorded the specific capacity changes of Ur–SA HEs-based RZIBs at different bending angles under −30 °C [65]. Shi et al. analyzed the capacity retentions of HEs-based RZIBs after bending with different angles at −70 °C [81]. Some works explain the design principles of HEs toward bending test at low temperatures: Fig. 11c reveals the hydrogel can be bendable under −30 °C because of the close contact between hydrogen bonds and gracile polymer chains, strengthening the mechanical stability; Fig. 11d illustrates the prepared principle of Ur–SA hydrogel is co-cross-linking between urea and SA, whose elasticity supports the bendability at many angles under the freezing temperatures; and Fig. 11e demonstrates the addition of salt and the optimization of H-bonds in the hydrogel makes HEs bend at different angles under extreme cold temperature.

#### Twisting Test

Twisting is one of the most prevalent and widely studied forms of deformation in research on HEs-based RZIBs. Compared to other deformation types, twisting often results in more severe damage to hydrogels. To address this, HEs must withstand the complex distortions and higher mechanical stresses induced by twisting, making twisting tests essential for evaluating their performance, especially at subzero temperatures.

Zhang et al. discovered the PAA hydrogel connected by the hydrogen bonds remained work after twisting deformations on RZIBs [104]. It was further found that hydrogen bonds contributed to strengthen cross-linking and improve flexibility of HEs to resist deformations. Lu et al. employed a para-polybenzimidazole (*p*-PBI)-based membrane as HEs in RZIBs, which could be twisted arbitrarily without performance fading after twisting [107]. Besides, some hydrogel species can be twisted without any damages at freezing temperatures. For instance, the conductive cellulose and AF HEs maintained a great performance under serious twisting at both room and ultra-low temperatures [39, 64], which can be attributed to the combination of flexible and anti-freezing design of HEs.

Hence, the design of hydrogels for twisting resistance requires careful optimization of the polymer structure. Modifying polymer chain chemistries is an effective technique to enhance the mechanical strength of hydrogel. Many hydrogel species are comprised of chains, such as PAA, PAM, and PVA. It is a transition from a two-dimensional shape to a three-dimensional model when hydrogel network formed. The hydrogel structure can be strengthened by extending polymer chains, thereby improving its structural integrity. Li et al. adopted this approach in their study (Fig. 7h): They cross-linked gelatin to prolong the original hydrogel chains, and results indicated the mechanical strength of the HEs had a huge improvement [53].

In summary, future studies will focus on designing HEs with enhanced flexibility to withstand severe deformations, including straining, compression, bending, and twisting. It is important to note that in RZIBs, only HEs can tolerate such deformations, while electrodes face significant limitations. These include the poor stretchability of metal anodes and cathodes, weak adhesion of active materials to current collectors, and delamination at the interface between HEs and electrodes under mechanical stress. Thus, the lack of flexibility in electrodes poses a major challenge for the development of flexible and wearable RZIBs. These challenges must be addressed in future battery designs.

### Harsh Damage

HEs-based RZIBs may experience damage from cutting, combustion, and water flooding during practical use, potentially leading to permanent battery failure. Recent research on flexible devices has extensively addressed battery safety against external damage, emphasizing the critical importance of self-healing, fireproof, and waterproof properties for the application of HEs-based RZIBs.

#### Cutting

Cutting is a frequent type of damage in flexible batteries, making the self-healing properties of HEs essential for their functionality. Numerous studies have been discussed the self-healing hydrogel in flexible devices with various configurations since it is striking to prolong the lifetime of energy storage devices and simultaneously lower the expense [50, 101]. The regulation and optimization of self-healing hydrogel is the major challenge for RZIB to maintain great performance under cutting [39].

**Fig. 12:**
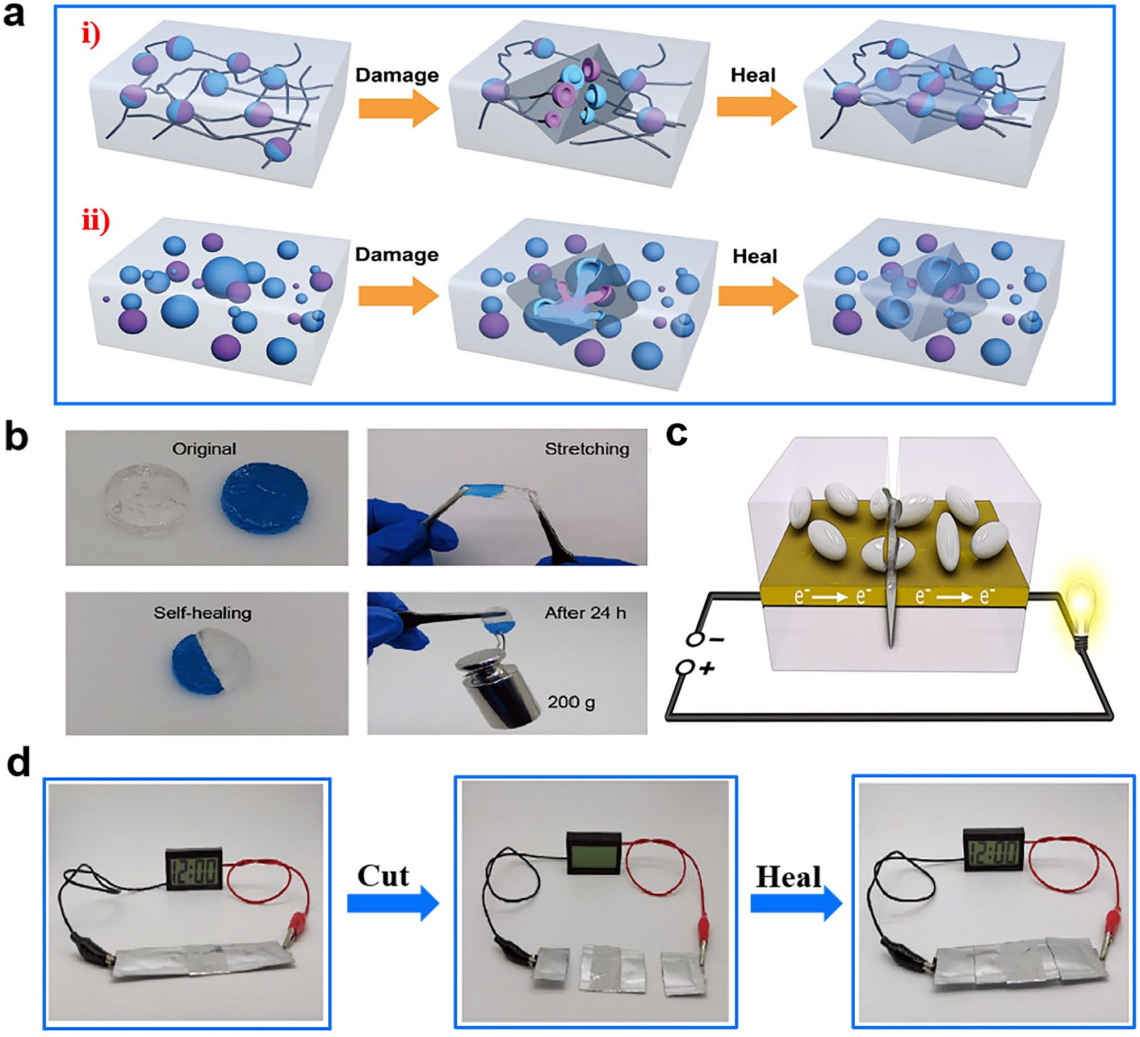
**a** Schematic diagram of self-healing hydrogel: (i) intrinsic self-healing polymers and (ii) extrinsic self-healing polymers. Reproduced with permission from Ref. [49]. Copyright 2017, Wiley–VCH. **b** Cut/self-healing test on PSBMA hydrogel. Reproduced with permission from Ref. [101]. Copyright 2020, Wiley–VCH. **c** Self-healing through exhaustion of healing agent embedded in a polymer microcapsule. Reproduced with permission from Ref. [108]. Copyright 2019, American Chemical Society. **d** Cut/self-healing test on quasi-solid-state Zn–MnO_2_ batteries based on carboxyl-modified PVA hydrogel. Reproduced with permission from Ref. [51]. Copyright 2019, American Chemical Society

Figure 12a elucidates the mechanism of self-healing hydrogel [49]. To date, its synthetic approaches can be categorized into two main types: (i) autonomous recovery of hydrogel relying on intrinsic reversible bonds; (ii) autonomous recovery through exhaustion of healing agents embedded in a polymer microcapsule. Three strategies are provided to design self-healing hydrogels according to type (i). Firstly, introducing reversible noncovalent linkages in hydrogels is feasible. From Fig. 12b, c, it can be observed the powerful self-healing capacities of PVA hydrogel with reversible noncovalent linkages. Li et al. modified PVA hydrogel by introducing carboxyl [51]. The carboxyl-based PVA hydrogel with salts of Zn(NO_3_)_2_ and MnSO_4_ was prepared by cross-linking of COO–Fe bonds, which showed PVA-based Zn–MnO_2_ batteries a good self-healing performance. Another modified PVA hydrogel cross-linked by hydrogen bonds was fabricated by Luo et al. [101]. Both these two species of PVA hydrogels are cross-linked by noncovalent between groups, the former was carboxyl, and the latter was hydroxyl. In this way, the batteries can autonomously recover their performances and continue to work after cutting. The second strategy is interdiffusion evolution between molecules. When damage occurs, the created crack surfaces have to rearrange due to the molecular discontinuity, and then, two crack surfaces have to be brought together and into contact. The third strategy is inducing a reversible Diels–Alder reaction. After cutting, the bonds between the Diels–Alder adduct are prone to be broken, since its bond strength is weaker than other covalent bonds, so the rearranged Diels–Alder reaction takes place. The second and third strategies, though typically, have rare applications in HEs-based RZIBs. Different from (i), the self-healing mechanisms of type (ii), can be obtained with the demand of exhaustion about materials pre-embedded in the fabrication. The often reported method for self-healing through exhaustion of healing agents is based on microcapsule fillers. The microcapsules are designed in Fig. 12d to be cracked by mechanical ruptures [108]. After cutting, the healing agents would be released from the microcapsules to heal the wound and fill the gap, so the HEs would be recovered by self-healing ability.

#### Burning

Flame resistance in batteries is increasingly critical for researchers and consumers, as spontaneous combustion incidents in electric devices and vehicles have raised safety concerns. Given that flammable batteries are likely to cause fires and explosions, fireproof battery materials are essential for reducing damaging risks [109, 110].

The conventional method adopted in batteries is adding flame retardants into the electrolytes to change its ignition point. This method is more common in LIBs with organic electrolytes. The organic electrolytes burn at relatively low temperatures, while HEs is hard to burn because water serves as the solvent in its polymer network. Diverse studies about HEs-based RZIBs reported the fireproofing performance. Some hydrogels species, e.g., guar gum, SA, PAM, and ion liquid, are nonflammable [36]. Using these species as HEs can effective prevent the burning of RZIBs. Under the heating from fire, these HEs-based RZIBs can maintain a stable electrochemical performance. Taking the PAM hydrogel as an example, it was reported that PAM HEs could exhibit great fire resistance in RZIBs and obtain a high capacity retention of 87.2% after exposing to the fire for 5 min [53].

To enhance safety, both nonflammable HEs and fireproof electrodes are essential in RZIBs to prevent fire or explosion under extreme conditions such as high temperatures or crashes. While the water solvent in hydrogels is safe, the hydrogen evolution reaction (HER) and H_2_ by-product generated from water splitting in HEs can increase the risk of battery combustion.

#### Soaking

The batteries may occasionally be exposed to splashes of water due to incautious operations in daily life, such as in a rainy environment. So the waterproofness of HEs-based RZIBs is a key factor for practical applications. Although waterproofness can be realized by exquisite packaging, it is more expected that the unpackaged battery can be waterproof to some extent. Zhi et al. reported a series of works to study the waterproofness of batteries in the previous works [43, 111]. They introduced the waterproof conception in pouch cells and tested the electrochemical performance of cells after soaking in the water. Subsequently, they developed no-package RZIBs comprised of Co_3_O_4-*x*_ cathode, PAM HEs, and the deposited Zn anode. To systematically investigate the waterproof performance, Ma et al. soaked and washed the assembled batteries in a glass vessel full of water. It was found that the batteries could remain a 98.6% open-circuit voltage retention. The yarn batteries were also researched and tested in the water, and it was observed that the batteries could maintain a capacity retention of 96.5%, even after continuous soaking for 12 h in water. Mo et al. explored the waterproof and low temperature-resisted performance by immersing hydrogel RZIBs in the ice bath during the same period [55]. The capacity retention was 89.3% after 5 min, demonstrating ice bath remained a large challenge for battery stability.

Besides, more attention should be paid to the swelling effect of HEs to investigate the waterproof ability. When soaking in water, the hydrogel becomes too bulk and soft to serve as electrolytes due to full absorption of water. In this case, the design idea should be oriented to the hydrogel with a high elastic modulus since a dense or high elastic network of hydrogel will not swell. Kim et al. proposed one kind of highly entangled PAM hydrogel network to resist swelling, whose swelling ratio could be controlled in a range of 100% ~ 105% [103].

HEs-based RZIBs are promising for wearable electronics due to their flexibility and energy density. However, since they may be exposed to wet or humid environments, such as rain, swimming, or sweating, the batteries must be meticulously designed to ensure reliable operation. Waterproof designs, therefore, hold significant potential for advancing wearable applications.

## Practical Applications

The study of various battery types aims to enable their practical use, with HEs-based RZIBs showing great promise for flexible and wearable electronics, especially under harsh conditions. Applications such as pouch batteries, fibrous batteries, and other configurations have drawn significant interest in the field of HEs-based RZIBs.

### Pouch Battery in Wearable Electronics Applications

**Fig. 13:**
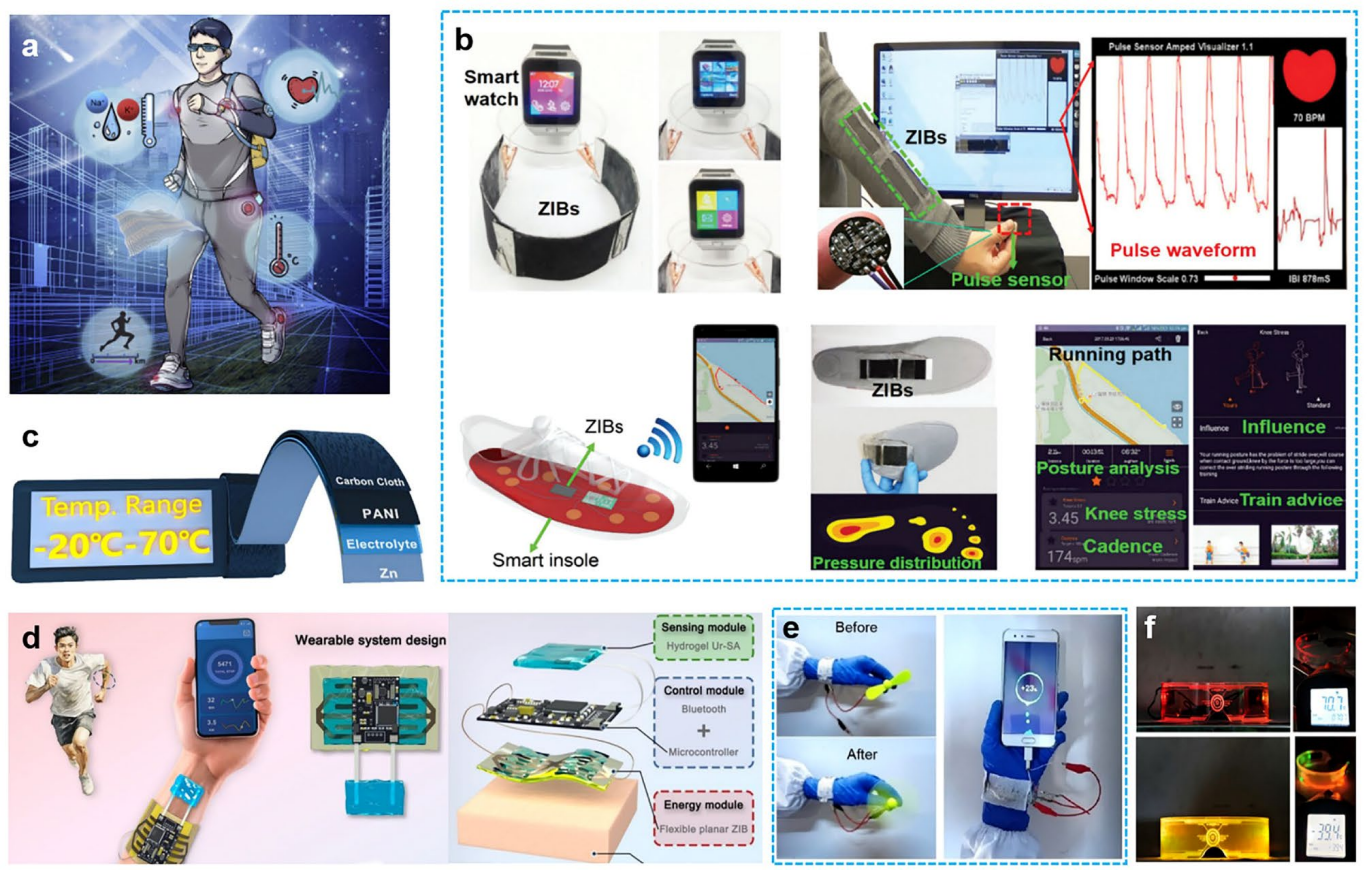
**a** Schematic illustration of the rechargeable smart textile and an autonomous body area electronics network based on functional HEs. Reproduced with permission from Ref. [112]. Copyright 2020, Elsevier. **b** Three RZIBs were connected in series to power a commercial smart watch; Four RZIBs were integrated in series to power a pulse sensor; Two ZIBs were connected in series to operate the smart insole; Optical photograph of the RZIBs-powered smart insole (top), the bending status of the smart insole (middle) and the pressure distribution when standing on the insole (bottom); and Running path (left) and the sport analysis results (right) obtained from the RZIB-powered smart insole. Reproduced with permission from Ref. [53]. Copyright 2018, Royal Society of Chemistry. **c** Illustration of the ZBL electrolyte-based ZIBs with extended lifespan across −20 to 70 °C. Reproduced with permission from Ref. [100]. Copyright 2024, Wiley–VCH. **d** Schematic illustrations of the biocompatible Ur–SA-based RZIBs: Ur-SA hydrogels are fabricated with the properties to apply to flexible planar cells, and the superior biocompatibility of Ur–SA as the sensor makes real-time physiological signals and human movements detection possible, powered by Ur–SA-based flexible planar cells. **e** Three series-connected fiber RZIBs can power a fan and charge a mobile phone when wearing on a human wrist. Reproduced with permission from Ref. [65]. Copyright 2024, Royal Society of Chemistry. **f** Demonstration of electronic devices powered by three pouch cells in series at −40 and 70 °C. Reproduced with permission from Ref. [82]. Copyright 2024, Nature Publishing Group

The pouch batteries have greater advantages on energy storage than fiber batteries. They are applied to various rechargeable smart textiles to monitor the body’s health and sports parameters (Fig. 13a) [112]. This kind of design firstly emerged in the study by Li et al., 2018. They used their HEs-based RZIBs in commercial smart watches, pulse sensors, smart insoles, etc., and measured the sensitive data (Fig. 13b) [53]. Three RZIBs were connected in series to power a commercial smart watch, four RZIBs were integrated in series to power a pulse sensor, and two ZIBs were connected in series to operate the smart insole. Optical photograph of the RZIBs-powered smart insole was presented with running path (left) and the sport analysis results (right). Liu et al. provided a basic structure of pouch batteries and operated the smartwatches at a wide temperature range in Fig. 13c [100]. It is illustrated the ZBL electrolyte-based ZIBs with extended lifespan across −20 to 70 °C. A flexible and biocompatible electronics is fabricated into Fig. 13d by Zhang et al., showing us the schematic illustrations of flexible electronics design [65]. Schematic illustrations of the biocompatible Ur–SA-based RZIBs are shown: Ur–SA hydrogels are fabricated with the properties to apply to flexible planar cells, and the superior biocompatibility of Ur–SA as the sensor makes real-time physiological signals and human movements detection possible, powered by Ur–SA-based flexible planar cells. Besides, the pouch RZIBs were also applied to wristbands, hat to support the power for the electronics (Fig. 13e, f) [19, 72]. Three series-connected fiber RZIBs can power a fan and charge a mobile phone when wearing on a human wrist. The electronic devices powered by three pouch cells in series are also demonstrated at −40 and 70 °C.

### Fiber Battery in Wearable Electronics Applications

**Fig. 14:**
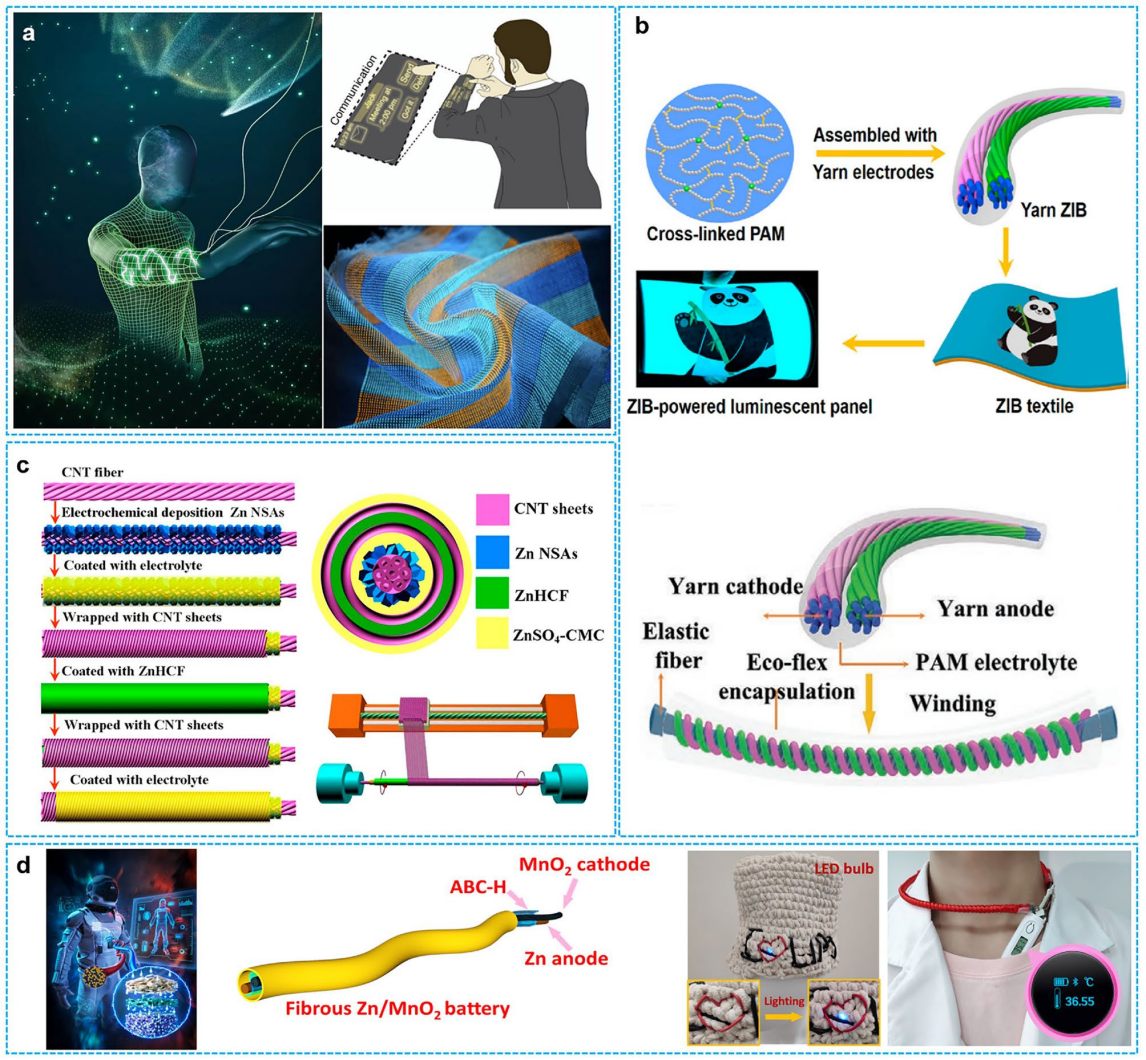
**a** Conceptual image shows that textiles integrated with a display and keyboard can be used as a communication tool for an application scenario of HEs-based RZIBs. Reproduced with permission from Ref. [113]. Copyright 2021, Nature Publishing Group. **b** Schematic diagram of fabrication and encapsulation of the yarn RZIBs based on HEs. Reproduced with permission from Ref. [45]. Copyright 2018, American Chemical Society. **c** Schematic illustrations showing the fabrication process of the CARZIB, its cross-section view, and wrapping ACNTSs around the modified CNTF. Reproduced with permission from Ref. [60]. Copyright 2019, American Chemical Society. **d** Wearable electronics application design of fibrous Zn–MnO_2_ batteries with tri-layer electrolytes. Reproduced with permission from Ref. [71] Copyright 2021, Wiley

The fibrous battery, as its name implies, mimics the morphology of textile fibers, exhibiting a high aspect ratio (slender and flexible form). This unique structure makes fibrous batteries a research hotspot in flexible and wearable electronics. Peng et al. developed a series of works about rechargeable textiles, cross-linked by fibrous batteries [113–115]. This conceptual textile design contributed to a potential communication tool with a functional raw material under complex deformations, including bending and twisting (Fig. 14a). This product was very close to commercial applications. Before these works, some specific concepts about fibrous batteries have been proposed. Li et al. [45] designed a fibrous battery by using double-helix yarn electrodes and a cross-linked PAM electrolyte. It was named “Yarn ZIB” (Fig. 14b) with high-performance, waterproof, tailorable, and stretchable properties. Zhang et al. [60] fabricated coaxial fiber aqueous rechargeable Zn-ion batteries (CARZIBs). This battery system adopted Zn nanosheet arrays (NSAs) on carbon nanotube fiber (CNTF) as the core electrode, ZnHCF composite on aligned CNT sheets (ACNTSs) as the outer electrode, and ZnSO_4_–CMC as the HEs (Fig. 14c). In our precious works [71, 116], we prepared fibrous Zn/MnO_2_ batteries using the tri-layer HEs. This fibrous battery was made into “Tai Chi” type (Fig. 14d), and the slimming hydrogel was separator to wrap the electrodes. Three kinds were relatively representative in fibrous RZIBs with HEs and were all multi-functional under harsh conditions [117, 118].

### Other Configurations in Wearable Electronics Applications

Flexible batteries with various configurations, such as paper-like, transparent, and stretchable designs, are being developed for wearable electronics [21, 23, 119, 120]. Paper-like batteries are fabricated using 3D-printed ultrathin hydrogel films (e.g., PAM and PVA), with thicknesses ranging from a few to tens of micrometers [121]. Transparent batteries, which integrate transparent HEs and electrodes, show great potential for applications in photoelectric conversion-storage devices and touch-sensitive systems [122]. Stretchable batteries, utilizing elastic HEs and electrodes, are key technologies in emerging fields, capable of withstanding twisting, bending, compression, and at least 1% integral stretching [123]. Beyond RZIBs, HEs also hold significant potential in other battery systems (e.g., LIBs, SIBs), as well as in solar cells, sensors, capacitors, and minireactors [122–126]. In the future, flexible RZIBs are expected to integrate with solar cells or other systems to maximize their advantages.

## Conclusions and Perspectives

**Fig. 15:**
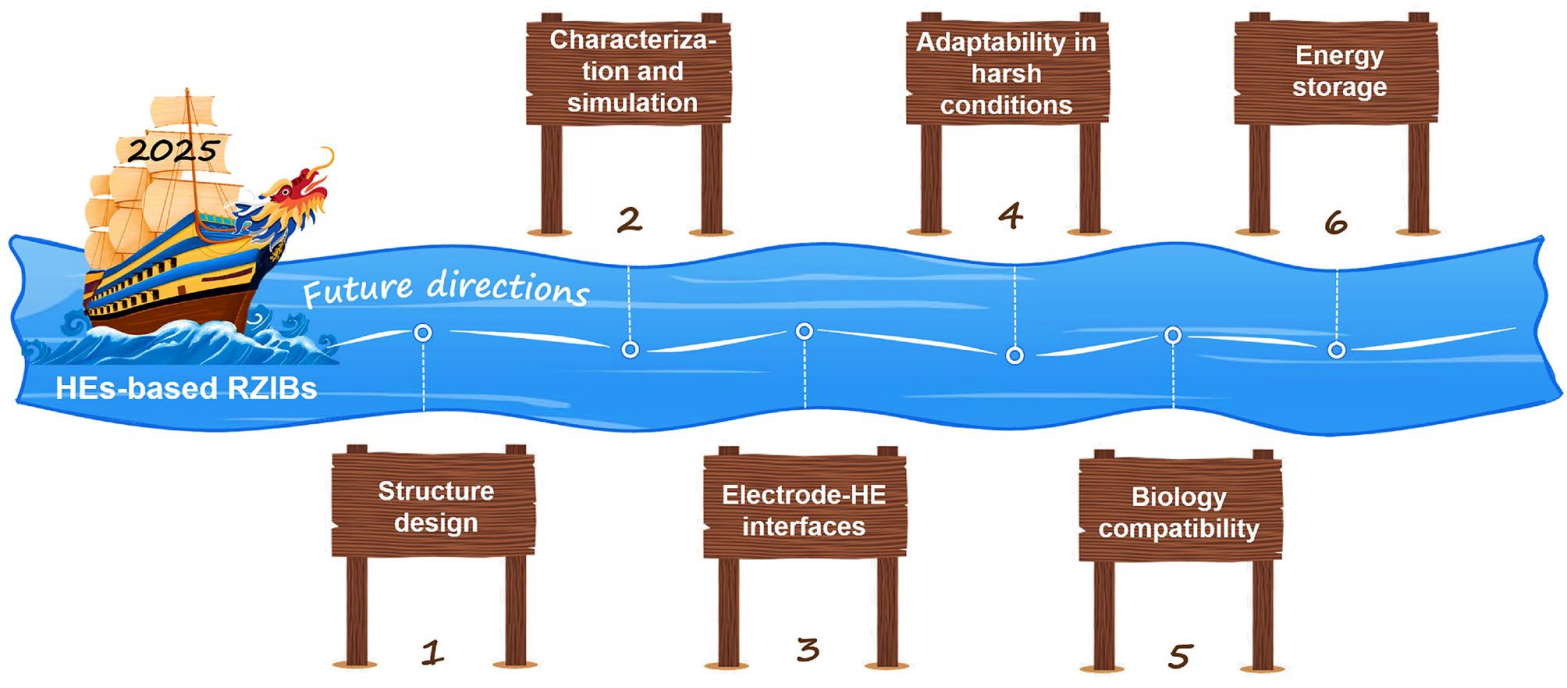
Schematic for six future directions of advanced HEs-based RZIBs: 1. Structure design; 2. Characterization and simulation; 3. Electrode-HE interfaces; 4. Adaptability in harsh conditions; 5. Biology compatibility; and 6. Energy storage

In conclusion, we have summarized the significant development of HEs-based RZIBs under harsh conditions. The large molecular polymers in hydrogels are recognized as the critical role in HEs. Albeit with satisfying properties in normal environments, they inevitably suffer from performance deterioration under harsh conditions. In this review, we first classify the hydrogel species as natural hydrogel, synthetic hydrogel, and hybrid hydrogel and further categorize them according to structures, functions, properties, and prepared approaches. Then, a brief history of HEs-based RZIBs is listed for a better understanding of recent advances. Subsequently, the current challenges of the interface compatibility between HEs and Zn anodes/cathodes are comprehensively discussed and multiple solutions are correspondingly presented. Most importantly, we emphasize the performances of HEs-assembled RZIBs under harsh conditions (i.e., high/low/wide temperature ranges, various mechanical deformations, soaking in water, burning in fire, and clipping) and provide the design strategies for building better HEs. However, some challenges still need to be addressed toward practical applications of HEs-based RZIBs. The remaining challenges and perspectives in this field are highlighted here (Fig. 15).

1. The structure design of hydrogel is the foundation for fabricating advanced HEs. The design strategies reported in HEs-based works mainly include monomers’ selection, cross-linking methods, and the design of additives, special groups, and multiple-layer structures. Monomers’ selection and cross-linking methods determine the functional groups and molecular connection types in hydrogel polymers, which can affect the intrinsic properties (e.g., stability, ion transfer, and mechanical strength) of HEs; functional additives (e.g., nanoparticles, zwitterions, and ionic liquids) and groups (e.g., C-N/F/O and O/S/N/P-H) can regulate the specific functions of HEs, involving the ion conductivity, temperature window, and interface compatibility, etc.; and the design of multiple-layer structures can manipulate the synergistic effects of HEs, involving reaction kinetics, storage capacity, cycling performance, rate capability, and Coulombic efficiency of RZIBs. To further improve the comprehensive performances of HEs for building better RZIBs, the profound exploration of new molecules and considerable insights into material structures are significantly required, including the assistance of theoretical calculation, database management, artificial intelligence (AI) selection.
2. Advanced characterization/simulation will be a new research trend in HEs-based RZIBs. Advanced instruments help researchers obtain reliable data of characterization and further explore the working mechanisms. Current works are mostly studied by ex situ technologies. However, in situ measuring methods are urgently demanded to present a more comprehensive investigation, e.g., in situ X-ray diffraction, photoelectron spectroscopy, Fourier-transform infrared spectrometer, Raman, etc. Moreover, it is encouraged to use a super connected system of precise instruments comprised of near-atmospheric X-ray photoelectron spectroscopy, photoemission electron microscopy, low energy electron microscopy, femtosecond laser, and atom force microscopy to achieve comprehensive results. Besides, theoretical simulation and calculation are pursued to have a profound understanding of HEs-based RZIBs from the perspective of molecule dynamics and thermodynamics. The simulation and calculation methods include typical density functional theory (DFT), Hartree-Fock method (HF), COMSOL simulation, which can be worked out by the software of Vienna Ab-initio Simulation Package (VASP), Material Studio (MS), Gaussian, and COMSOL Multiphysics.
3. Optimizing the interfaces between HEs and cathodes/anodes plays a crucial role in enabling the practical applications of flexible RZIBs. Currently, most HEs only stabilize the interface by direct physical interactions, which is insufficient to address the growth of Zn dendrites and the dissolution of cathode materials. Therefore, novel strategies are focused on designing compatible materials and functional hydrogel structures, as well as minimizing by-products and suppressing side reactions at the interfaces. For the HEs–Zn anode interface, the selection and design of HEs are primarily aimed at creating a protective layer for the Zn anode. The –CONH_2_ functional group of HEs can induce uniform Zn deposition because its strong interaction to Zn^2+^. The lean-water and rich-OH design in HEs can suppress HER and Zn corrosion because the reduced water dosage and the H-bond formation between HEs and H_2_O. In addition, more attention should be paid to the effects of HEs on the Zn^2+^ desolvation behavior, SEI formation/structure, and electric double layer. For the HEs–cathode interface, suitable modifications of HEs can improve the reversibility and kinetics reaction of the cathode. For example, the rich-Mn^2+^/H^+^ design in HEs can stabilize the MnO_x_ cathode and promote the convention of Mn^4+^/Mn^2+^ redox. The poly-cations design in HEs can avoid the polyiodide shuttle effect to improve the stability of I_2_ cathode.
4. Regarding the urgent demands for HEs-based RZIBs under harsh conditions, oriented design of hydrogel species will consequentially be one major direction in the future. We have discussed the performances and design principles of HEs for extreme temperatures, serious deformations, and harsh damages. In future works, the super high/low temperatures, puncturing, and knotting will be further studied for constructing better HEs-based RZIBs. Moreover, the tolerance of HEs under high working current/voltage is also a research direction among harsh conditions. Besides, the HEs-based RZIBs may synchronously suffer from two or more serious damages under multiple harsh conditions, which should also be considered for practical applications.
5. Biology compatibility becomes a new design direction for HEs to well meet large-scale wearable applications. Biopolymer materials, such as sodium alginate, polyaspartic acid, gelatin, and cellulose, generally originate from plants or animals. However, these natural biocompatible materials are barely used as single-component HEs because of their fragile features. They usually combine with synthetic hydrogel species to prepare the hybrid HEs to strengthen mechanical properties. In order to address the challenges encountered by natural hydrogel materials, it is desirable to find novel natural hydrogel species with multiple functions, for example, directly developing hyperelastic species to avoid the fracture of HEs; exploiting eco-friendly species to address the pollution from HEs; introducing conductive species to improve the ion conductivity of HEs. In addition, novel natural hydrogel species with new functions of photosensitivity, thermal sensitivity, or sound sensitivity should be developed to meet the requirements of “smart batteries” in health monitoring and personal care in wearable applications.
6. The HEs are engineered to enhance the energy storage performance of RZIBs. Many works report the potential of HEs-based RZIBs in flexible and wearable devices for safe energy storage, e.g., rechargeable wristbands, Bluetooth earphones, and self-thermal clothes. Note that these works on the performance evaluation of batteries are most based on laboratory-made cells, from which the results may not be straightforwardly translated into practical batteries. In the future, the industrial fabrication or commercial process will be highlighted for the applications of batteries. Testing conditions to meet industrial requirements should be considered from aspects such as high mass loading, minimal electrolyte usage, and optimized cathode-to-anode ratio. In the future, the HEs-based RZIBs also hold significant promise for the application in high-safety electric vehicles designed for short-distance travel.
